# Genistein Pretreatment Attenuates Ovalbumin-Induced Food Allergy in Mice with Intestinal Barrier Preservation and Modulation of Gut Microbiota and Metabolites

**DOI:** 10.3390/foods15111995

**Published:** 2026-06-03

**Authors:** Xiaomei Yi, Wen Deng, Kuan Gao, Xiaoying Ou, Keyu Tang, Qian Zeng, Yuanyuan Ni, Xiaohui Liang, Zhihua Wu, Yong Wu, Yanhai Xie, Hongbing Chen, Anshu Yang

**Affiliations:** 1 Sino-German Joint Research Institute, Nanchang University, Nanjing Dong Lu 235, Nanchang 330047, China; yi1291022@163.com (X.Y.); 13340115395@163.com (W.D.); 367900240001@email.ncu.edu.cn (K.G.); oxying130@163.com (X.O.); keyutang0712@163.com (K.T.); zq2831164447@163.com (Q.Z.); 15608168281@163.com (Y.N.); liangxiaohui0727@163.com (X.L.); wuzhihua@ncu.edu.cn (Z.W.); ericyo918@hotmail.com (Y.W.); xieyh818@ncu.edu.cn (Y.X.); chenhongbing@ncu.edu.cn (H.C.); 2 State Key Laboratory of Food Science and Resources, Nanchang University, Nanjing Dong Lu 235, Nanchang 330047, China; 3 School of Food Science and Technology, Nanchang University, Nanchang 330047, China; 4 International Institute of Food Innovation, Nanchang University, Nanchang 330200, China

**Keywords:** food allergy, genistein, nutraceuticals, intestinal barrier, gut microbiota, short-chain fatty acids, fecal metabolomics

## Abstract

Food allergy (FA) is an increasing public health concern, highlighting the urgent need for safe, bioactive-based preventive strategies. This study evaluated genistein, a plant-derived isoflavone, in an ovalbumin (OVA)-induced murine FA model. Genistein was administered before sensitization and throughout allergy induction. Clinical symptoms, rectal temperature, diarrhea, OVA-specific antibodies, mast cell responses, intestinal barrier markers, gut microbiota, short-chain fatty acids (SCFAs), and fecal metabolites were assessed using immunological, histological, microbiome, and metabolomic analyses. Genistein pretreatment prevented OVA-induced clinical symptom scores, rectal temperature decline, diarrhea occurrence, OVA-specific antibody responses, and mast cell responses. These changes were accompanied by preservation of jejunal tight junction-related markers and modulation of T-cell-associated immune responses. In vitro, genistein modulated antigen uptake, maturation-associated features of bone marrow-derived dendritic cells (BMDCs), and BMDC-driven CD4^+^ T-cell polarization. In parallel, genistein-pretreated mice showed altered gut microbial structure, higher relative abundances of selected SCFA-associated taxa, increased fecal butyrate, and fecal metabolomic alterations involving purine metabolism, bile-acid-related metabolism, and tryptophan-related microbial metabolites. Consistently, correlation analyses indicated associations among microbial taxa, metabolites, immune indicators, and intestinal barrier markers. Together, these findings provide preliminary mechanistic insight into genistein in experimental FA and support further investigation of genistein as a dietary bioactive candidate for FA prevention.

## 1. Introduction

Food allergy (FA) has become an increasingly important public health concern worldwide, affecting both patients and caregivers and imposing substantial medical, dietary, and psychosocial burdens [1]. Recent studies have further highlighted its socioeconomic impact, including increased healthcare utilization, food-related out-of-pocket expenses, reduced quality of life, and caregiver productivity loss, underscoring the need for effective preventive and management strategies [2,3,4]. FA is generally characterized by aberrant immune responses to otherwise harmless dietary proteins, most commonly immunoglobulin E (IgE)-mediated hypersensitivity [5]. Clinical manifestations may range from mild gastrointestinal or cutaneous symptoms to severe systemic reactions [6]. At present, FA management still relies predominantly on allergen avoidance and treatment of accidental exposure [7]. However, strict avoidance is difficult to maintain in real-life dietary settings and does not address the mechanisms underlying immune sensitization and impaired oral tolerance [8,9]. Therefore, increasing attention has been directed toward safe dietary approaches based on bioactive compounds that may help limit allergic sensitization and support mucosal homeostasis.

The pathogenesis of FA is complex and involves coordinated disturbances in mucosal immunity and intestinal barrier function [10,11]. A T helper 2 (Th2)-skewed immune response promotes allergen-specific IgE responses, mast cell activation, and the release of inflammatory mediators [12]. In addition to this classical allergic pathway, other immune components, including Th17-associated responses and regulatory T-cell-related pathways, may contribute to intestinal inflammation, immune imbalance, and epithelial dysfunction [13,14]. The intestinal epithelium serves as both a physical barrier and an immunologically active interface between luminal antigens and host tissues. When epithelial integrity is compromised, increased allergen passage across the mucosa may further promote sensitization and downstream allergic responses [15,16,17]. Accordingly, strategies that modulate mucosal immune responses while preserving epithelial barrier integrity are of particular relevance in FA prevention research.

Increasing evidence highlights the important role of the gut microbiota and its metabolites in regulating immune tolerance and epithelial homeostasis [18,19]. Gut microbial dysbiosis has been linked to increased allergic susceptibility [20], whereas microbiota-derived metabolites, including short-chain fatty acids (SCFAs), tryptophan-derived indole metabolites, and bile-acid-related molecules, are increasingly recognized as mediators of host–microbe interactions [21,22,23]. Among these metabolites, butyrate has been implicated in epithelial barrier maintenance and immune regulation [24]. These observations suggest that dietary bioactives capable of influencing gut microbiota–metabolite profiles and mucosal homeostasis may represent promising nutraceutical candidates for FA prevention.

Genistein is a naturally occurring isoflavone with reported anti-inflammatory, antioxidant, and immunomodulatory properties [25,26,27]. Previous studies have shown that genistein can influence inflammatory signaling, epithelial barrier-related responses, and gut microbial composition in different experimental contexts [28,29,30]. However, its preventive role in FA remains insufficiently characterized, particularly with respect to the integrated relationship among mucosal immune responses, intestinal barrier function, gut microbiota, and microbial metabolites. Moreover, whether genistein affects dendritic cell-related antigen handling and downstream T-cell-associated responses under allergen-stimulated conditions requires further clarification. Therefore, the present study evaluated the preventive effects of genistein in an ovalbumin (OVA)-induced murine model of FA. By integrating clinical assessment, immunological analysis, intestinal barrier evaluation, gut microbiota profiling, SCFA profiles, and untargeted fecal metabolomics, this study aimed to examine whether genistein pretreatment could influence the development of OVA-induced allergic responses and whether any observed effects were associated with changes in gut microbiota–metabolite profiles and intestinal barrier-related markers. In addition, bone marrow-derived dendritic cell (BMDC) experiments were performed to assess the effects of genistein on antigen uptake, maturation-related phenotypes, and downstream T-cell-associated responses under OVA-stimulated conditions. Although purified genistein rather than biomass-derived preparations was used in the present work, these findings may provide preliminary mechanistic information for future studies evaluating genistein-containing botanical materials or standardized preparations.

## 2. Materials and Methods

### 2.1. Materials and Reagents

Genistein (purity > 99%), PEG-300, and OVA peptide (OVA323–339, sequence: ISQAVHAAHAEINEAGR, purity 99.98%, Cat. No. HY-P0286) were obtained from MedChemExpress (Monmouth Junction, NJ, USA). The OVA peptide corresponds to residues 323–339 of ovalbumin and was used as a defined T-cell epitope in the relevant in vitro assays. Ovalbumin (OVA, ≥98%) and aluminum hydroxide (alum, ≥98%) were purchased from Sigma-Aldrich (St. Louis, MO, USA). Antibodies for immunoglobulin detection were obtained from SouthernBiotech (Birmingham, AL, USA). Flow cytometry antibodies against T-bet, GATA3, Foxp3, CD11c, CD86, CD103, MHC-II, CD40, and CD80 were purchased from BioLegend (San Diego, CA, USA), and anti-RORγt was obtained from BD Biosciences (San Jose, CA, USA). Primary antibodies against Claudin-1 and Occludin were purchased from Abcam (Cambridge, MA, USA), and anti-ZO-1 was obtained from Proteintech Group, Inc. (Rosemont, IL, USA). Corresponding secondary antibodies were purchased from Thermo Fisher Scientific (Waltham, MA, USA). ELISA kits for cytokines and mouse mast cell protease-1 (mMCP-1) were obtained from Thermo Fisher Scientific. RPMI 1640 medium and fetal bovine serum (FBS) were purchased from Solarbio (Beijing, China) and Cellmax (Beijing, China), respectively. Recombinant granulocyte-macrophage colony-stimulating factor (GM-CSF) and interleukin-4 (IL-4) were obtained from PeproTech (Cranbury, NJ, USA). Cell Counting Kit-8 (CCK-8) reagent was purchased from GLPBIO (Montclair, CA, USA). HiScript III RT SuperMix (+gDNA wiper) and ChamQ Universal SYBR qPCR Master Mix were obtained from Vazyme Biotech Co., Ltd. (Nanjing, China). Detailed information on antibodies, ELISA kits, catalog numbers, and supplier information is provided in Supplementary Table S1.

### 2.2. Animal Experiments

#### 2.2.1. Animal Model and Experimental Design

An OVA-induced FA mouse model was established based on a previously reported protocol [31] with minor modifications. Female BALB/c mice (5 weeks old, *n* = 40) were obtained from GemPharmatech (Nanjing, China) and housed under specific pathogen-free conditions at 23 ± 2 °C and 40–60% relative humidity under a 12 h light/dark cycle, with free access to food and water. An egg-free and soy-free AIN-93G diet was supplied by Shuyu Biotechnology Co., Ltd. Female mice were selected to maintain consistency with established OVA-induced FA protocols and because previous studies have reported greater susceptibility of female mice to OVA-induced FA, including more severe anaphylactic responses and greater impairment of intestinal barrier function [32]. After a 1-week acclimatization period, mice were randomly assigned to five groups (*n* = 8 per group): Control, OVA, low-dose genistein (L-Gen), medium-dose genistein (M-Gen), and high-dose genistein (H-Gen). The individual mouse was considered the experimental unit. Gen was dissolved in PEG-300/sterile saline (1:1, *v*/*v*). Mice in the L-Gen, M-Gen, and H-Gen groups received Gen at 5, 10, and 20 mg/kg body weight/day, respectively, by oral gavage at a fixed volume of 200 μL per mouse per day. Mice in the Control and OVA groups received an equal volume of vehicle. The selected Gen doses were based on previous in vivo studies and preliminary tolerability considerations [33,34].

Gen or vehicle administration began 7 days before the first sensitization and continued throughout the experimental period. On days 0 and 14, mice in the OVA and Gen-pretreated groups were sensitized by intraperitoneal injection of OVA (50 μg) adsorbed to alum (1 mg) in 250 μL sterile saline. From day 28, mice were orally challenged every other day for a total of six challenges. OVA-sensitized mice received OVA challenge solution containing 20 mg OVA in 250 μL phosphate-buffered saline (PBS), whereas Control mice received the corresponding PBS challenge. Fecal samples were collected 1 h after the final oral challenge and stored at −80 °C until analysis. After the final assessment, mice were euthanized under deep anesthesia. Blood, cecal contents, jejunal tissues, spleens, and other required samples were collected. Serum was separated for antibody and mMCP-1 assays, whereas cecal contents and tissue samples were snap-frozen in liquid nitrogen and stored at −80 °C until further analysis. An additional 8-week-old female BALB/c mouse was used for bone marrow-derived dendritic cell (BMDC) generation. Naïve CD4^+^ T cells were isolated from DO11.10 transgenic mice (C.Cg-Tg(DO11.10)10Dlo/J, The Jackson Laboratory, Bar Harbor, ME, USA). All animal procedures were approved by the Animal Ethics Committee of Nanchang University (Approval No. NCULAE-20250903001) and were conducted in accordance with the ARRIVE guidelines and the NIH Guide for the Care and Use of Laboratory Animals.

#### 2.2.2. Assessment of Allergic Symptoms and Diarrhea

After each oral challenge, mice were monitored for 60 min for clinical allergic signs, general condition, and diarrhea occurrence. Clinical allergic symptoms were scored according to a previously described scoring system [35,36], and the detailed criteria are provided in Supplementary Table S2. Fecal morphology was evaluated using predefined scoring criteria [37] as shown in Supplementary Table S3. Mice with a fecal score of 2 or 3 were considered to have diarrhea. Diarrhea occurrence was recorded after oral challenge, and the diarrhea rate was calculated as the percentage of mice meeting the diarrhea criterion within each group. Rectal temperature was measured 30 min after challenge using a digital thermometer (BAT-12; Physitemp Instruments LLC, Clifton, NJ, USA). For standardized endpoint comparisons among groups, clinical allergic symptom scores, fecal scores, and rectal temperature measured after the final oral challenge were used for statistical analysis unless otherwise indicated.

### 2.3. Measurement of Serum Ova-Specific Antibodies and Mmcp-1

Blood samples were collected one day after the final oral challenge and allowed to clot at room temperature for 2 h. Serum was separated by centrifugation at 4000 rpm for 20 min and stored at −80 °C before analysis. Serum OVA-specific IgE, IgG, IgG1, and IgG2a levels were measured by indirect ELISA. Briefly, 96-well plates were coated overnight at 4 °C with OVA at 50 μg/mL and then blocked with 3% gelatin at 37 °C for 1 h. After washing, diluted serum samples were added and incubated at 37 °C for 1 h. Serum samples were diluted 1:10,000 for OVA-specific IgE and IgG2a detection and 1:200,000 for OVA-specific IgG and IgG1 detection. After washing, plates were incubated with the appropriate secondary antibodies, including biotinylated goat anti-mouse IgE or horseradish peroxidase-conjugated goat anti-mouse IgG, IgG1, and IgG2a antibodies, at a dilution of 1:5000. Streptavidin-HRP was further applied for IgE detection. TMB was used as the chromogenic substrate, and the reaction was stopped with 2 M H_2_SO_4_. Absorbance was measured at 450 nm using a microplate reader (Model 3001, Cat. No. 5250030; Thermo Fisher Scientific, Waltham, MA, USA). Serum mMCP-1 was quantified using a commercial ELISA kit according to the manufacturer’s instructions.

### 2.4. Evaluation of Immune Responses

#### 2.4.1. Flow Cytometric Analysis of Splenic CD4^+^ T Cell Subsets

Single-cell suspensions were prepared from spleens by mechanical dissociation followed by red blood cell lysis. Cells were stained with fluorescent antibodies against surface CD4 and CD25 and then fixed, permeabilized, and stained intracellularly for T-bet, GATA3, RORγt, and Foxp3 using a fixation/permeabilization kit according to the manufacturer’s instructions. Data were acquired using a CytoFLEX flow cytometer (Beckman Coulter, Inc., Brea, CA, USA) and analyzed with FlowJo software v10.8.1 (FlowJo LLC, Ashland, OR, USA).

#### 2.4.2. Cytokine Secretion by Splenocytes

Splenocytes were cultured in complete RPMI 1640 medium at a density of 5 × 10^6^ cells/mL and stimulated with OVA at 200 μg/mL for 72 h. Culture supernatants were collected, and concentrations of IL-4, IL-5, IL-13, IL-17A, IFN-γ, and IL-10 were measured using commercial ELISA kits according to the manufacturer’s instructions.

#### 2.4.3. Functional Analysis of BMDCs and T Cell Responses

Bone marrow-derived dendritic cells (BMDCs) were generated from the femurs and tibias of 8-week-old female BALB/c mice and cultured in complete RPMI 1640 medium supplemented with GM-CSF (20 ng/mL) and IL-4 (10 ng/mL). To evaluate cytotoxicity, BMDCs were treated with Gen (0, 12.5, 25, 50, or 100 μM) for 24 h, and cell viability was assessed using a CCK-8 assay according to the manufacturer’s instructions.

For antigen uptake analysis, BMDCs were pretreated with Gen and then incubated with FITC-labeled OVA for 4 h at 37 °C. For phenotypic analysis, BMDCs were stimulated with OVA peptide and stained for surface markers related to antigen presentation and maturation, including CD86, MHC-II, CD40, CD80, and CD103. To evaluate downstream T-cell responses, naïve CD4^+^ T cells isolated from DO11.10 mice were co-cultured with antigen-pulsed BMDCs at a BMDC: T-cell ratio of 1:10 for 72 h. After incubation, T cell differentiation-associated markers, including T-bet, GATA3, RORγt, and Foxp3, were analyzed by flow cytometry.

### 2.5. Intestinal Tissue Analysis

#### 2.5.1. Intestinal Morphology, Mast Cell Infiltration, and Barrier-Related Proteins

Jejunal segments were collected, fixed in 4% paraformaldehyde for 24 h, embedded in paraffin, and sectioned at 4 μm. After deparaffinization and rehydration, sections were stained with hematoxylin and eosin (H&E) to evaluate intestinal morphology.

Mast cell infiltration was assessed by immunohistochemical staining for tryptase. Briefly, jejunal sections were incubated with rabbit anti-tryptase antibody (E7M2U, 1:100; #19523, CST), followed by an HRP-conjugated secondary antibody. Signals were visualized using diaminobenzidine (DAB), and nuclei were counterstained with hematoxylin. Images of H&E-stained sections and tryptase immunohistochemistry were captured under a light microscope at 200× magnification. Tryptase-positive mast cells were defined as DAB-positive cells located in the jejunal lamina propria. Mast-cell infiltration was quantified by counting tryptase-positive cells in non-overlapping fields at 200× magnification. For each mouse, multiple non-overlapping fields were analyzed, and the average number of tryptase-positive mast cells per field was used for statistical analysis.

The localization and abundance of tight junction-related proteins, including Claudin-1, Occludin, and ZO-1, were examined by immunofluorescence staining. After antigen retrieval, sections were permeabilized, blocked, and incubated overnight at 4 °C with primary antibodies against Claudin-1, Occludin, and ZO-1, followed by fluorescence-labeled secondary antibodies. Nuclei were counterstained with DAPI. Immunofluorescence images were acquired using an Olympus BX53 fluorescence microscope (Olympus Corporation, Hachioji, Tokyo, Japan) at 400× magnification.

#### 2.5.2. RNA Extraction and Quantitative Real-Time PCR

Total RNA was extracted from jejunal tissues using TRIzol reagent according to the manufacturer’s instructions. RNA concentration and purity were assessed before reverse transcription. cDNA was synthesized using HiScript III RT SuperMix (+gDNA wiper). Quantitative real-time PCR was performed using ChamQ Universal SYBR qPCR Master Mix on a QuantStudio 3 Real-Time PCR System (Applied Biosystems, Thermo Fisher Scientific, Waltham, MA, USA). Relative gene expression was calculated using the 2^−ΔΔCt^ method and normalized to *GAPDH* as the housekeeping gene. Primer sequences are listed in Supplementary Table S4.

### 2.6. Microbiota and Metabolite Analysis

#### 2.6.1. 16S rRNA Sequencing

Cecal contents were collected immediately after sacrifice, snap-frozen in liquid nitrogen, and stored at −80 °C until analysis. Microbial DNA was extracted using the MagPure Stool DNA KF Kit B (Magen, Guangzhou, China) according to the manufacturer’s instructions and purified using a KingFisher Flex system (Thermo Fisher Scientific, Waltham, MA, USA). The V3-V4 hypervariable region of the 16S rRNA gene was amplified using primers 338F (5′-ACTCCTACGGGAGGCAGCAG-3′) and 806R (5′-GGACTACHVGGGTWTCTAAT-3′) and sequenced on the DNBSEQ-G400 platform (BGI-Shenzhen, China). Data analysis was conducted using the BGI Meta platform (http://meta.bgi.com).

#### 2.6.2. Quantification of Short-Chain Fatty Acids (SCFAs)

Fecal SCFAs were quantified by UHPLC-MS/MS after derivatization with 3-nitrophenylhydrazine. Briefly, 50 mg of fresh fecal sample was mixed with 400 μL of methanol/acetonitrile extraction solvent (2:1, *v*/*v*) in the presence of stainless-steel magnetic beads and homogenized at 50 Hz for 120 s. After bead removal, the homogenate was centrifuged at 20,000× *g* for 3 min at 4 °C. To facilitate protein precipitation, 60 μL of pre-cooled methanol/acetonitrile solution (2:1, *v*/*v*) was added to the supernatant, followed by vortexing for 5 min and incubation at −20 °C for 4 h. Samples were then centrifuged at 20,000× *g* for 15 min at 4 °C, and the clarified supernatant was used for derivatization.

For derivatization, 40 μL of supernatant was sequentially mixed with 20 μL of 3-nitrophenylhydrazine and 20 μL of 1-(3-dimethylaminopropyl)-3-ethylcarbodiimide. The mixture was vortexed for 1 min and incubated for 30 min. After derivatization, 80 μL of 10% aqueous acetonitrile was added and thoroughly mixed. A stable isotope-labeled internal standard mixture containing ^13^C-labeled acetate, propionate, butyrate, isobutyrate, isovalerate, valerate, and caproate was added at a fixed amount to each sample, calibration standard, and quality control sample. Subsequently, 90 μL of the derivatized solution was diluted with an equal volume of ultrapure water, passed through a 0.22 μm membrane filter, and centrifuged at 3000× *g* for 5 min at 4 °C before UHPLC-MS/MS analysis.

Chromatographic separation was achieved on a Waters ACQUITY UPLC I-Class Plus system (Waters Corporation, Milford, MA, USA) using a BEH C18 column (2.1 mm × 50 mm, 1.7 µm). Solvent A was water containing 0.1% formic acid, and solvent B was acetonitrile containing 0.1% formic acid. The gradient program was as follows: 0–1.00 min, 15% B; 1.00–4.00 min, 55% B; 4.00–5.00 min, 55% B; 5.00–5.10 min, 15% B; and 5.10–6.00 min, 15% B. The flow rate was 0.35 mL/min, and the column temperature was maintained at 40 °C. Detection was performed on a SCIEX QTRAP 6500+ triple-quadrupole mass spectrometer (SCIEX, Framingham, MA, USA) operated in positive electrospray ionization mode using scheduled multiple reaction monitoring.

#### 2.6.3. Untargeted Metabolomics Analysis

Untargeted fecal metabolomics was performed on a predefined subset of six biological samples per group. Samples were selected before LC-MS analysis based on sufficient fecal sample availability, acceptable sample integrity, and complete corresponding phenotypic data. Fecal samples were thawed on ice, and 25 mg aliquots were extracted with 800 µL pre-chilled methanol:acetonitrile:water (2:2:1 *v*/*v*/*v*) containing 10 μL of an internal standard mixture and ceramic beads. The internal standard mixture consisted of d3-Leucine, ^13^C9-Phenylalanine, d5-Tryptophan, and ^13^C3-Progesterone and was added before extraction to monitor sample preparation and LC-MS analytical stability. Samples were homogenized at 50 Hz for 5 min using a tissue grinder, ultrasonicated for 10 min and incubated at −20 °C for 1 h. The homogenates were centrifuged at 25,000× *g* for 15 min, and the supernatants were collected and lyophilized. Dried extracts were reconstituted in 600 μL of methanol:water (1:9, *v*/*v*), vortexed for 1 min, ultrasonicated for 10 min, and centrifuged again at 25,000× *g* for 15 min. The final supernatant was filtered through a 0.22 µm membrane and transferred to autosampler vials for analysis. Quality control samples were prepared by pooling equal volumes from each sample extract and were injected periodically throughout the analytical batch to monitor instrument stability and reproducibility. Raw data were processed using the BGI Metabolomics Platform (https://biosys.bgi.com) for peak detection, alignment, and normalization. Differentially expressed metabolites were defined using fold changes (FC) ≥ 1.2, variable importance in projection (VIP) ≥ 1.0, and *p* < 0.05. Correlation analyses among microbial genera, metabolites, immune indicators, and intestinal barrier-related markers were performed using OmicsStudio (https://www.omicstudio.cn/tool, accessed on 10 May 2026).

### 2.7. Statistical Analysis

Data are presented as means ± SEM. Statistical analyses were performed using GraphPad Prism 8.0. Differences among groups were analyzed using one-way analysis of variance (ANOVA) followed by Tukey’s multiple comparison test. Correlation analyses were performed using Spearman’s rank correlation coefficient. All experimental groups, including Control, OVA, and Gen-pretreated groups, were included in the statistical comparisons. A value of *p* < 0.05 was considered statistically significant.

## 3. Results

### 3.1. Effects of Genistein Pretreatment on Allergic Responses and Humoral Immunity

**Figure 1:**
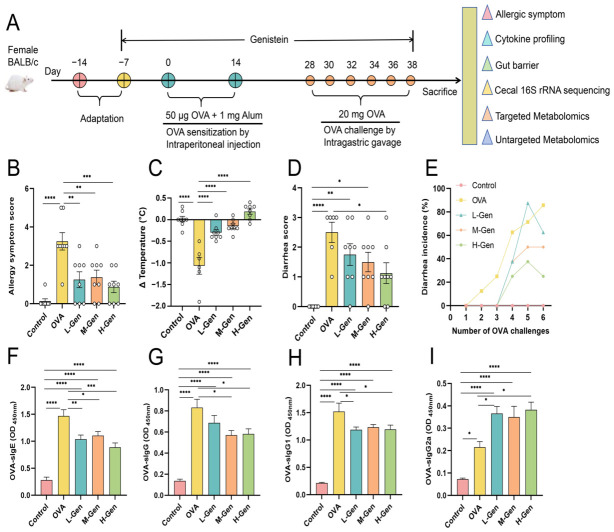
Genistein (Gen) pretreatment prevented OVA-induced food allergy (FA) symptoms and modulated OVA-specific antibody responses. (**A**) Experimental timeline for OVA sensitization/challenge and Gen administration. The colored triangle symbols in panel A indicate different detection categories. (**B**) Clinical symptom score after the final OVA challenge. (**C**) Changes in rectal temperature following the final challenge. (**D**) Diarrhea severity scores. (**E**) Diarrhea incidence. (**F**–**I**) Levels of OVA-specific IgE, IgG, IgG1 and IgG2a in serum. Data are presented as means ± standard error of the mean (SEM). Each circle represents an individual mouse. Statistical significance: * *p* < 0.05, ** *p* < 0.01, *** *p* < 0.001, **** *p* < 0.0001.

To evaluate the effects of genistein (Gen) pretreatment on OVA-induced allergic responses, an OVA-induced murine model was established (Figure 1A). Following the final oral challenge, OVA-sensitized mice exhibited elevated clinical symptom scores (Figure 1B, *p* < 0.0001, vs. Control group) and a marked decrease in rectal temperature (Figure 1C, *p* < 0.0001, vs. Control group). Gen pretreatment partially limited these OVA-induced responses. Compared with the OVA group, Gen-pretreated mice showed lower clinical symptom scores (Figure 1B), and the OVA-induced reduction in rectal temperature was less pronounced after Gen administration (Figure 1C, *p* < 0.0001). In addition, diarrhea severity and incidence showed a decreasing trend in Gen-pretreated groups compared with the OVA group, with the most apparent reduction observed in the H-Gen group (Figure 1D,E).

Consistent with these clinical observations, Gen pretreatment was associated with differences in antigen-specific humoral immune responses at the endpoint of the experiment. Serum OVA-specific IgE levels were markedly higher in the OVA group than in the Control group, whereas Gen-pretreated mice exhibited significantly lower OVA-specific IgE levels than the OVA group, with the H-Gen group showing an approximately 40% lower level relative to the OVA group (Figure 1F, *p* < 0.001). OVA-specific IgG levels were also lower in the Gen-pretreated groups than in the OVA group, particularly in the M-Gen and H-Gen groups (Figure 1G, *p* < 0.05, vs. OVA group). In addition, OVA-specific IgG1 levels were lower, whereas OVA-specific IgG2a levels were higher, in the Gen-pretreated groups than in the OVA group, with significant differences observed in the indicated groups (Figure 1H,I, *p* < 0.05).

### 3.2. Genistein-Associated T-Cell Responses and Cytokine Profiles

**Figure 2:**
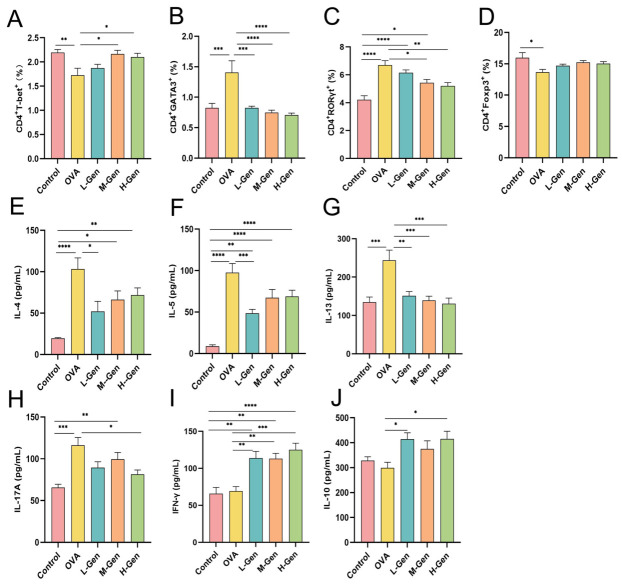
Gen pretreatment modulated splenic CD4^+^ T-cell subsets and cytokine production in OVA-sensitized mice. (**A**–**D**) Frequencies of splenic CD4^+^T-bet^+^ (Th1), CD4^+^GATA3^+^ (Th2), CD4^+^ RORγt^+^ (Th17), and CD4^+^ Foxp3^+^ (Treg) cells. (**E**–**J**) Concentrations of IL-4, IL-5, IL-13, IL-17A, IFN-γ, and IL-10 in splenocyte culture supernatants. Data are presented as means ± SEM. Statistical significance: * *p* < 0.05, ** *p* < 0.01, *** *p* < 0.001, **** *p* < 0.0001.

To further investigate the immune changes associated with Gen pretreatment, splenic T-cell subsets were analyzed by flow cytometry, and jejunal T-cell-related immune signatures were evaluated by measuring lineage-associated transcription factors and cytokines. In splenic CD4^+^ T cells, the proportion of CD4^+^ T-bet^+^ cells was higher in the M-Gen and H-Gen groups than in the OVA group (Figure 2A and Figure S1, *p* < 0.05). OVA sensitization was associated with increased proportions of CD4^+^ GATA3^+^ Th2-related cells and CD4^+^ RORγt^+^ Th17-related cells, whereas these OVA-induced changes were lower after Gen pretreatment (Figure 2B,C and Figure S1). The proportion of CD4^+^ Foxp3^+^ regulatory T-cell-related cells was reduced in the OVA group compared with the Control group (Figure 2D and Figure S1, *p* < 0.05). Gen pretreatment prevented this decrease, with splenic CD4^+^ Foxp3^+^ cell proportions in the Gen-pretreated groups comparable to those in the OVA group; however, these differences did not reach statistical significance (Figure 2D).

In jejunal tissues, OVA challenge decreased the mRNA expression of *T-bet* and *Foxp3* and increased the mRNA expression of *GATA3* and *RORγt*, accompanied by higher *IL-4* and *IL-5* mRNA levels compared with the Control group (Figure S2A–F). Gen pretreatment partially limited these OVA-induced transcriptional changes. Compared with the OVA group, *T-bet* and *Foxp3* mRNA levels were higher in all Gen-pretreated groups, whereas *GATA3* expression was lower. *RORγt* and *IL-5* mRNA levels were significantly reduced in the H-Gen group, while *IL-4* expression was lower in the M-Gen and H-Gen groups compared with the OVA group (Figure S2A–F). No significant difference in *IFN-γ* mRNA expression was observed between the Control and OVA groups or between the OVA and Gen-pretreated groups (Figure S2G).

Cytokine secretion was further assessed in culture supernatants of OVA-stimulated splenocytes. OVA sensitization increased the production of IL-4, IL-5, IL-13, and IL-17A compared with the Control group (Figure 2E–H). Gen pretreatment differentially affected cytokine secretion among dose groups. Compared with the OVA group, IL-4 and IL-5 levels were significantly lower in the L-Gen group, IL-13 levels were reduced in all Gen-pretreated groups, and IL-17A levels were significantly lower in the H-Gen group (Figure 2E–H). IFN-γ and IL-10 levels did not differ significantly between the Control and OVA groups. However, IFN-γ levels were higher in all Gen-pretreated groups compared with the OVA group, and IL-10 levels were significantly increased in the L-Gen and H-Gen groups (Figure 2I,J).

### 3.3. Genistein-Associated Jejunal Barrier Markers and Mast Cell Responses

**Figure 3:**
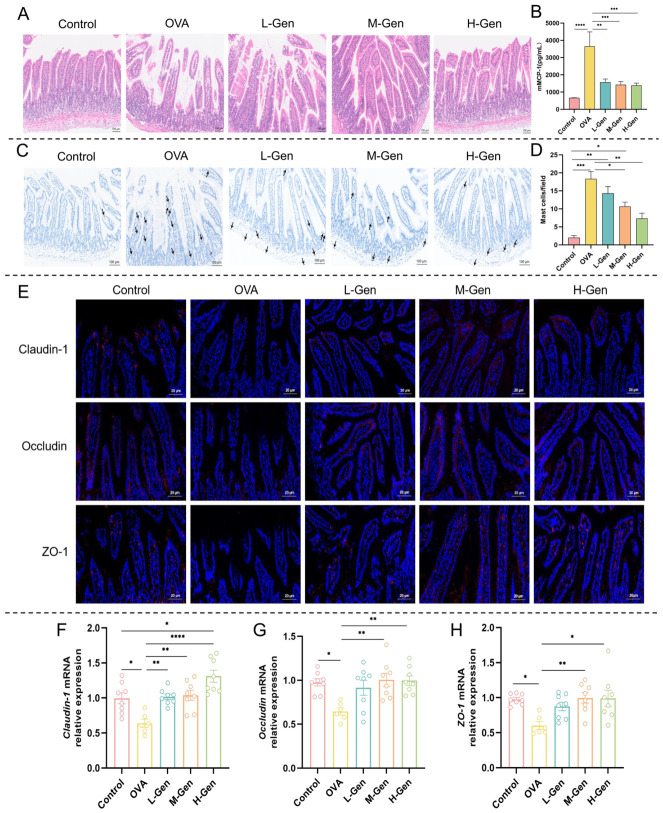
Gen pretreatment preserved jejunal barrier-associated features and reduced mast-cell-associated responses. (**A**) Representative H&E staining of jejunal sections. (**B**) Levels of mouse mast cell protease-1 (mMCP-1) in serum. (**C**) Representative tryptase immunostaining of jejunal sections (200× magnification). Arrows indicate tryptase-positive mast cells, and magnified views highlight individual mast cells for clarity. (**D**) Quantification of jejunal mast cells. (**E**) Immunofluorescence staining of tight junction proteins (Claudin-1, Occludin, and ZO-1) in the jejunal epithelium. (**F**–**H**) mRNA expression levels of *Claudin-1*, *Occludin*, and *ZO-1* in jejunal tissue. Data are presented as means ± SEM. Each circle represents an individual mouse jejunal tissue sample. Statistical significance: * *p* < 0.05, ** *p* < 0.01, *** *p* < 0.001, **** *p* < 0.0001.

To determine whether Gen pretreatment was associated with changes in intestinal barrier integrity, jejunal tissues were further examined. Representative H&E-stained sections showed that Control mice exhibited well-organized villus architecture and an intact epithelial structure (Figure 3A). In contrast, OVA-sensitized mice displayed villus shortening and epithelial disruption. In Gen-pretreated mice, the representative H&E sections showed less apparent OVA-associated morphological disruption than that observed in the OVA group (Figure 3A).

Mast cell-related responses were then evaluated to further characterize mucosal allergic inflammation. Serum mouse mast cell protease-1 (mMCP-1) levels were significantly elevated in OVA-treated mice compared with Control mice (Figure 3B, *p* < 0.0001). Gen pretreatment resulted in lower serum mMCP-1 levels compared with the OVA group (Figure 3B). Consistently, tryptase immunohistochemistry showed increased accumulation of tryptase-positive mast cells in the jejunum of OVA-sensitized mice, whereas Gen pretreatment was associated with lower mast cell abundance, particularly in the M-Gen and H-Gen groups (Figure 3C,D).

Tight junction-related proteins were further examined to assess intestinal barrier-associated changes. OVA sensitization reduced the expression of Claudin-1, Occludin, and ZO-1, whereas Gen pretreatment partially preserved their expression patterns (Figure 3E). This observation was further supported at the transcriptional level. Compared with the Control group, the OVA group showed lower mRNA expression of *Claudin-1*, *Occludin*, and *ZO-1* (Figure 3F–H, *p* < 0.05). *Claudin-1* mRNA levels were higher in all Gen-pretreated groups relative to the OVA group, whereas *Occludin* and *ZO-1* mRNA levels were significantly higher in the M-Gen and H-Gen groups relative to the OVA group (Figure 3F–H).

### 3.4. Effects of Genistein on BMDC Phenotypes and T-Cell Responses In Vitro

To complement the in vivo findings, an in vitro bone marrow-derived dendritic cell (BMDC) model was used to evaluate the effects of Gen on dendritic cell function and subsequent T-cell-associated responses. CCK-8 analysis showed that Gen did not markedly reduce BMDC viability at concentrations up to 50 μM (Figure S3). Therefore, concentrations of 12.5–50 μM were used in subsequent experiments.

**Figure 4:**
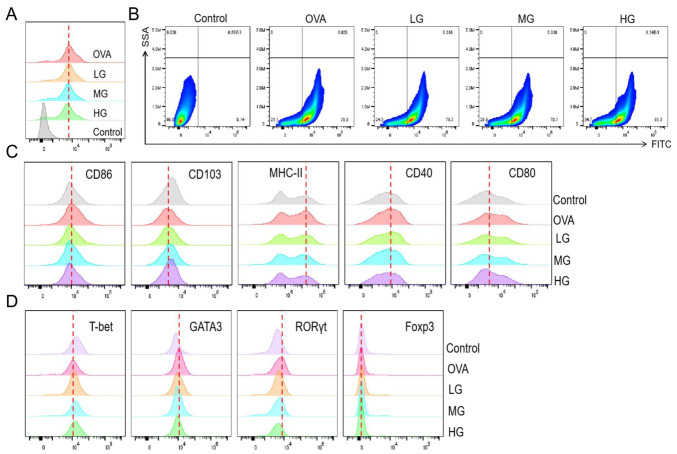
Gen modulated antigen uptake, maturation-associated features of BMDCs, and BMDC-driven CD4^+^ T-cell polarization in vitro. (**A**) Representative histogram of FITC-OVA uptake by BMDCs. (**B**) Representative flow cytometry dot plots of FITC-OVA uptake by BMDCs. (**C**) Surface expression of co-stimulatory markers (CD86, CD103, MHC-II, CD40, and CD80) on BMDCs. (**D**) Representative histogram of CD4^+^ T-cell subsets in BMDC–T cell co-cultures (Th1/T-bet^+^, Th2/GATA3^+^, Th17/RORγt^+^, and Treg/Foxp3^+^). Dashed red lines indicate the peak positions of the OVA group for comparison, and different colors represent different experimental groups.

Antigen uptake by BMDCs was assessed using FITC-labeled OVA. Incubation with FITC-OVA resulted in detectable antigen uptake. Quantitative flow cytometry analysis indicated that the proportion of FITC-positive BMDCs was lower in Gen-treated groups than in the OVA-only group (Figure 4A,B). The maturation-associated phenotype of BMDCs was evaluated by flow cytometry based on the frequencies of surface marker-positive cells. Representative panels of CD86, MHC-II, CD40, CD80, and CD103 expression are shown in Figure 4C and Figure S4. In these panels, OVA stimulation appears to increase the percentages of CD86^+^, MHC-II^+^, CD40^+^, and CD80^+^ BMDCs compared with the Control group, whereas Gen-treated BMDCs appear lower in these populations compared with the OVA group. In contrast, CD103^+^ BMDCs appear higher in the Gen-treated groups compared with the OVA group (Figure 4C and Figure S4).

To assess the functional consequences of these phenotypic changes, BMDCs were co-cultured with naïve CD4^+^ T cells. Downstream T-cell-associated responses were evaluated by flow cytometry using T-bet, GATA3, RORγt, and Foxp3 as markers related to Th1-, Th2-, Th17-, and regulatory T-cell-associated phenotypes, respectively. Representative flow cytometry panels of CD4^+^ T cell subsets (T-bet, GATA3, RORγt, and Foxp3) are shown in Figure 4D and Figure S5. In these panels, antigen-pulsed BMDCs appear to increase the percentages of GATA3^+^ and RORγt^+^ CD4^+^ T cells compared with the Control group, whereas Gen-treated BMDCs appear lower in these populations compared with the OVA group. In contrast, T-bet^+^ and Foxp3^+^ CD4^+^ T cells appear higher in the corresponding Gen-treated BMDC co-cultures compared with the OVA group (Figure 4D and Figure S5).

### 3.5. Genistein-Associated Gut Microbiota Composition and Fecal SCFA Profiles

**Figure 5:**
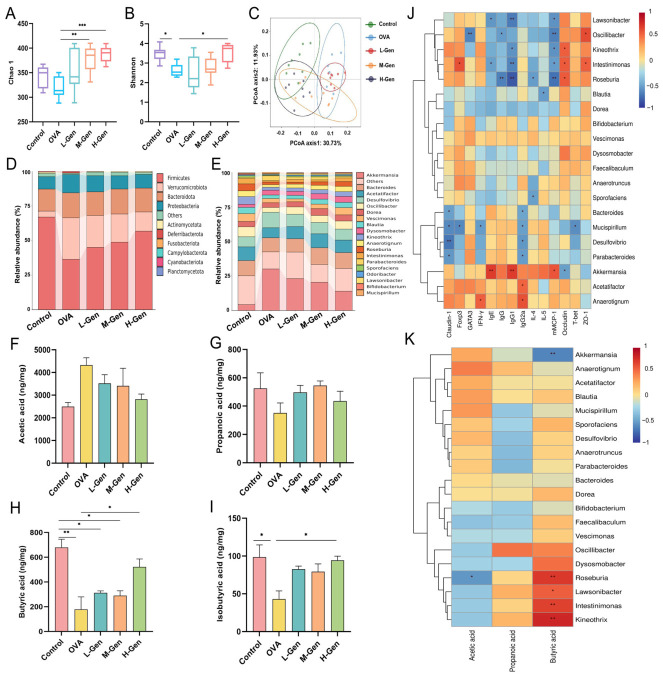
Gen pretreatment altered gut microbiota composition, SCFAs production, and microbiota–host associations. (**A**) Chao1 richness index. (**B**) Shannon diversity index. (**C**) PCoA of β-diversity. (**D**,**E**) Relative abundance of gut microbiota at the phylum (**D**) and genus (**E**) levels. (**F**–**I**) Fecal concentrations of acetate, propionate, butyrate, and isobutyrate. (**J**,**K**) Spearman correlation heatmaps: (**J**) microbial genera versus host-related parameters; (**K**) microbial genera versus SCFAs. Red indicates positive correlations and blue indicates negative correlations. Data are presented as means ± SEM. Statistical significance: * *p* < 0.05, ** *p* < 0.01, *** *p* < 0.001, non-significant comparisons are not shown.

To determine whether Gen pretreatment was associated with changes in the intestinal microbiota, 16S rRNA sequencing was performed using cecal contents. Good’s coverage exceeded 0.99 across all groups, indicating sufficient sequencing depth (Figure S6A). Alpha-diversity analysis showed differences in microbial richness and diversity among groups. Shannon, observed species, and ACE indices differed significantly between the OVA and Control groups (Figure 5B and Figure S6B,C, *p* < 0.05). Compared with the OVA group, Chao1 and observed species differed significantly in the M-Gen and H-Gen groups (Figure 5A and Figure S6B), Shannon differed significantly in the H-Gen group (Figure 5B), and ACE differed significantly in all Gen-pretreated groups (Figure S6C). Principal coordinate analysis (PCoA) showed apparent separation between the OVA and Control groups, whereas the H-Gen group was positioned closer to the Control group (Figure 5C), indicating a shift in microbial community structure after Gen pretreatment.

At the phylum level, the OVA group showed apparent changes in the relative proportions of major bacterial phyla compared with the Control group, including a lower relative abundance of *Firmicutes* and higher relative abundances of *Proteobacteria* and *Verrucomicrobiota*. Gen pretreatment partially shifted these phylum-level patterns toward those observed in Control mice (Figure 5D). At the genus level, Gen pretreatment was associated with lower relative abundance of *Desulfovibrio* and *Akkermansia* and higher relative abundances of *Blautia*, *Roseburia*, and *Intestinimonas* (Figure 5E). These compositional changes were accompanied by differences in fecal short-chain fatty acid (SCFA) profiles. Acetate and propionate did not show statistically significant differences among the relevant groups (Figure 5F,G). In contrast, butyrate and isobutyrate levels were lower in the OVA group than in the Control group and were higher in the H-Gen group compared with the OVA group (Figure 5H,I).

Correlation analysis was performed to examine associations among gut microbial genera, allergic indicators, immune-related markers, and barrier-related parameters. SCFA-associated genera showing higher relative abundance after Gen pretreatment, including *Roseburia* and *Intestinimonas*, were negatively correlated with allergic indicators such as mMCP-1 and positively correlated with *Occludin* expression (Figure 5J). In contrast, *Akkermansia* showed positive correlations with allergic indices and negative associations with *Occludin* in this model (Figure 5J). Additional correlations between bacterial genera and fecal SCFAs are shown in Figure 5K. Fecal butyrate was positively correlated with *Roseburia*, *Lawsonibacter, Intestinimonas*, and *Kineothrix*, and negatively correlated with *Akkermansia. Roseburia* also showed a negative correlation with acetate. These correlation results describe associations and should not be interpreted as evidence of causality.

### 3.6. Genistein-Associated Fecal Metabolic Profiles and Host–Microbe Associations

Untargeted fecal metabolomics was performed to characterize metabolic changes associated with Gen pretreatment. Based on the consistent changes observed in clinical and immunological parameters, the H-Gen group was selected for in-depth metabolomic analysis. This approach complemented targeted SCFA analysis by capturing broader metabolic alterations associated with Gen pretreatment.

**Figure 6:**
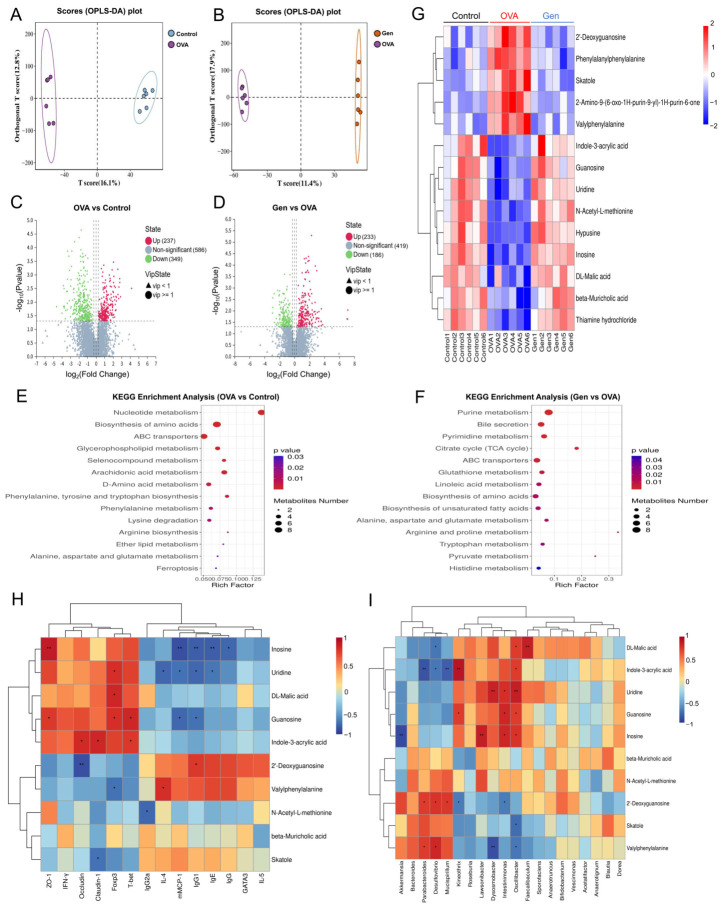
Gen pretreatment altered the fecal metabolome and microbiota–metabolite–host associations. (**A**,**B**) OPLS-DA scores plot for OVA vs. Control (**A**) and Gen vs. OVA (**B**). (**C**,**D**) Volcano plots of differential metabolites (OVA vs. Control; Gen vs. OVA). (**E**,**F**) KEGG pathway enrichment analysis (OVA vs. Control; Gen vs. OVA). (**G**) Heatmap of key differential metabolites. (**H**,**I**) Spearman correlation heatmaps: (**H**) key metabolites versus host-related parameters; (**I**) microbial genera versus metabolites. Red indicates positive correlations and blue indicates negative correlations. Statistical significance: * *p* < 0.05, ** *p* < 0.01.

Orthogonal projections to latent structures discriminant analysis (OPLS-DA) showed group-level separation between the Control and OVA groups (Figure 6A), as well as between the OVA and Gen (H-Gen) groups (Figure 6B), indicating distinct fecal metabolic profiles among these groups. Differential analysis identified widespread metabolite changes following OVA exposure (Figure 6C), whereas Gen pretreatment was associated with 233 upregulated and 186 downregulated metabolites compared with the OVA group (Figure 6D). KEGG pathway analysis showed that OVA sensitization affected pathways related to nucleotide metabolism, amino acid biosynthesis, ABC transporters, and arachidonic acid metabolism (Figure 6E). In contrast, Gen pretreatment was associated with changes in pathways related to purine metabolism, bile secretion, and tryptophan metabolism (Figure 6F).

Key metabolites contributing to group separation were further examined (Figure 6G). Compared with Controls, the OVA group displayed lower levels of indole-3-acrylic acid, guanosine, uridine, inosine, and β-muricholic acid. After Gen pretreatment, these metabolite levels partially shifted toward those observed in Control mice. Conversely, OVA exposure increased the levels of 2′-deoxyguanosine, phenylalanylphenylalanine, and valylphenylalanine, whereas these metabolites were lower in the Gen group compared with the OVA group (Figure 6G).

Correlation analysis revealed associations between fecal metabolites and host response-related parameters. Tight junction markers, including *Occludin* and *Claudin-1*, were positively correlated with indole-3-acrylic acid, whereas *Occludin* showed a negative association with metabolites elevated in the OVA group, such as 2′-deoxyguanosine. In addition, IgE and mMCP-1 were negatively correlated with inosine and uridine (Figure 6H), supporting an association between fecal metabolite profiles and allergic severity-related indicators.

Further integration of microbiota and metabolomic data revealed coordinated associations between specific microbial taxa and fecal metabolites (Figure 6I). Genera showing higher relative abundance in the H-Gen group, such as *Oscillibacter* and *Kineothrix*, were positively correlated with indole-3-acrylic acid and guanosine. In contrast, taxa showing higher relative abundance in the OVA group, such as *Desulfovibrio*, showed positive correlations with 2′-deoxyguanosine and valylphenylalanine and negative associations with indole-3-acrylic acid (Figure 6I). Together, these results suggest that H-Gen pretreatment was associated with coordinated changes in gut microbiota composition, fecal metabolite profiles, and host response-related parameters in experimental FA.

## 4. Discussion

Food allergy (FA) is increasingly recognized as a multifactorial disorder involving dysregulated type 2 immune responses, impaired intestinal barrier function, and altered gut microbial homeostasis [38,39]. In the present study, Gen pretreatment was associated with partial limitation of OVA-induced allergic manifestations and was accompanied by coordinated changes in immune responses, intestinal barrier-related markers, gut microbiota composition, and fecal metabolic profiles. These findings suggest that Gen may be associated with changes in interconnected immune, epithelial barrier, and host–microbe metabolic pathways, although the causal relationships among these processes remain to be clarified.

The lower allergic responses observed in Gen-pretreated mice were accompanied by changes in humoral immune responses. OVA-specific IgE and mMCP-1 levels were lower in Gen-pretreated mice than in OVA mice, and the IgG1/IgG2a balance shifted toward a less Th2-dominant pattern. These changes are biologically relevant as allergen-specific IgE is a central mediator of immediate allergic reactions through mast cell activation [40,41], whereas the IgG1/IgG2a ratio is commonly used to reflect Th2- versus Th1-associated immune tendencies [42,43]. Therefore, the humoral immune changes observed in Gen-pretreated mice suggest that the response associated with Gen was not limited to changes in individual allergic markers, but was also related to partial attenuation of the Th2-skewed immune imbalance induced by OVA sensitization. This interpretation is consistent with previous reports showing that flavonoids and other plant-derived bioactives from different structural families may influence food allergy-related responses through modulation of antibody-associated immune responses, Th2-skewed inflammation, epithelial barrier function, and gut microbiota-related pathways [44,45,46,47]. Nevertheless, these findings should be interpreted as immunological associations rather than direct evidence that Gen controls antibody class switching in vivo.

The regulation of T-cell-associated responses further supports this interpretation. In the present model, OVA sensitization was associated with a Th2/Th17-skewed immune state, whereas Gen pretreatment reduced Th2- and Th17-related features and was accompanied by changes in regulatory or counter-regulatory immune signals. This pattern is important as IL-4, IL-5, and IL-13 are central mediators of IgE production, mast cell-associated responses, and amplification of allergic inflammation [12,13,48]. At the same time, the reduction in IL-17A-related responses suggests that the response associated with Gen was not restricted to classical Th2-related changes, but may also involve inflammatory pathways linked to mucosal injury and epithelial dysfunction [49,50]. The Foxp3-related findings were interpreted according to the tissues and methods used. Splenic regulatory T-cell-related responses were assessed by flow cytometry, whereas jejunal *Foxp3* was measured at the mRNA expression level. Therefore, these results should be considered complementary rather than directly comparable. The jejunal *Foxp3* mRNA data suggest that Gen pretreatment was associated with local immune-related changes at the intestinal mucosal interface, where tolerance to dietary antigens is established and maintained [51,52]. Taken together, these observations indicate that Gen pretreatment was associated with immune rebalancing in experimental FA at both systemic and local levels. This view is also in agreement with earlier studies showing that plant-derived flavonoids can attenuate Th2-dominant allergic inflammation while supporting regulatory responses at barrier sites [53,54,55]. These immunological changes likely contribute to the observed mitigation of OVA-induced clinical and serological responses.

Preservation of intestinal barrier-associated features appears to be another important component of the response associated with Gen. The intestinal epithelium functions not only as a physical barrier but also as an active immunological interface that controls antigen access and shapes downstream immune responses [56]. Once barrier integrity is disrupted, luminal allergens can more readily cross the epithelium and reinforce both sensitization and effector activation. In the present study, Gen pretreatment was associated with less apparent jejunal morphological damage, partial preservation of tight-junction-related markers, and reduced mast-cell-associated mucosal responses. These findings suggest that Gen may help maintain epithelial resilience under allergic conditions. Such barrier preservation could potentially limit allergen translocation and reduce continued stimulation of mucosal immune pathways [57,58]. The concurrent reduction in mMCP-1 and jejunal mast cell accumulation further supports a close relationship between epithelial barrier maintenance and attenuation of allergic effector activation. Similar barrier-supportive actions have been reported for other plant-derived polyphenols and microbiota-related metabolites, further supporting the biological plausibility of this mechanism [59,60]. However, the present study cannot determine whether barrier preservation was a primary effect of Gen or a secondary consequence of reduced allergic inflammation.

The BMDC experiments provide additional mechanistic support for the possibility that Gen may influence FA at the level of antigen-presenting cell regulation. Dendritic cells are pivotal in allergic sensitization as they govern antigen uptake, processing, and presentation, thereby shaping downstream T-cell differentiation [61,62,63]. In this study, Gen treatment appeared to reduce FITC-OVA uptake by BMDCs and was associated with a lower proportion of BMDCs expressing maturation-associated markers, while CD103^+^ BMDCs appeared more abundant. Although CD103 expression alone does not establish tolerogenic function, CD103^+^ dendritic cells are commonly associated with more tolerogenic features in the intestinal immune system [64], suggesting that Gen may favor a less inflammatory antigen-presenting profile. The co-culture results further indicated that Gen-treated BMDCs appeared to reduce Th2- and Th17-like T-cell-associated responses and were more compatible with immune rebalancing. Although this in vitro system cannot fully reproduce the complexity of the intestinal microenvironment, it nevertheless provides mechanistic support for the possibility that Gen modulates allergic sensitization through dendritic cell–T-cell interactions. This interpretation aligns with previous studies showing that phytochemicals can influence dendritic cell function and downstream adaptive immune responses [65,66,67]. These in vitro findings provide mechanistic support for the in vivo observations but require further validation in complex intestinal contexts.

Beyond host immune-related responses, alterations in gut microbiota composition appeared to represent another component of the Gen-associated phenotype in this model. Increasing evidence indicates that allergic susceptibility is closely linked to microbial composition and to the capacity of the gut ecosystem to support mucosal homeostasis [68]. In the present study, Gen pretreatment partially shifted the microbial community toward the profile observed in Control mice. Of particular interest were the higher relative abundances of *Blautia*, *Roseburia*, and *Intestinimonas*, genera often associated with fermentative metabolism and SCFA-related metabolic capacity [69]. This microbial pattern is biologically relevant as butyrate is known to support epithelial barrier integrity, regulate inflammatory signaling, and promote tolerogenic immune responses [70,71]. Correlation analyses further supported this interpretation, showing that SCFA-associated genera with higher relative abundance after Gen pretreatment were negatively associated with allergic indices and positively associated with tight-junction markers and *Foxp3* expression. These coordinated relationships suggest a plausible microbiota–metabolite–host axis involved in the response to Gen. This interpretation is also consistent with current evidence linking reduced butyrate availability to impaired immune tolerance and increased susceptibility to FA [24,72]. Nevertheless, these associations should be interpreted cautiously, because the present study was not designed to establish direct causality between specific microbial taxa, SCFAs, and attenuation of allergic responses. The interpretation of individual microbial taxa should also remain context-dependent. Although *Akkermansia* is frequently regarded as beneficial in metabolic and mucosal contexts [73,74], its role may vary across disease settings, host conditions, and microbial environments [75]. In the present OVA-induced FA model, *Akkermansia* showed positive correlations with allergic indices and negative associations with Occludin. This does not indicate that *Akkermansia* is universally detrimental. Rather, it suggests that its expansion in this specific allergic context may reflect a disease-associated microbial configuration. Therefore, the microbiota-related findings in the present model are better understood as context-dependent and mechanistically supportive rather than universally generalizable.

The metabolomic results further extend this mechanistic framework by showing that Gen pretreatment was associated with broader remodeling of host–microbe co-metabolism. Compared with approaches focusing only on immune phenotypes or microbial composition, the combination of targeted SCFA analysis and untargeted fecal metabolomics provides a wider view of intestinal changes during allergic inflammation. Pathway analysis of the H-Gen versus OVA comparison indicated associations with changes in several metabolic pathways, including purine metabolism, bile secretion, and tryptophan metabolism-related pathways. However, these results should not be interpreted as direct evidence that Gen specifically regulates these pathways. Untargeted metabolomics provides useful hypothesis-generating information, whereas pathway-specific conclusions require targeted validation. Among the altered metabolites, indole-3-acrylic acid, a tryptophan-related indole metabolite, is noteworthy because microbial indole derivatives have been linked to epithelial protection and mucosal immune regulation, partly through aryl hydrocarbon receptor-related signaling [76]. Therefore, the present metabolomic findings suggest the possible involvement of tryptophan-related microbial metabolic changes. However, receptor activation, targeted metabolite quantification, and metabolite intervention experiments were not performed, so this mechanism remains to be confirmed. The partial shift in β-muricholic acid levels also suggests a possible contribution of bile-acid-related metabolism, which is increasingly recognized as an important regulator of host immunity and gut microbial ecology [77,78]. In addition, the partial shift in inosine and uridine toward Control-associated levels may be relevant to inflammatory regulation and tissue homeostasis [79,80]. Conversely, metabolites elevated in the OVA group, including 2′-deoxyguanosine and peptide-like metabolites, were lower after H-Gen pretreatment. Although their specific roles in FA require further clarification, their correlations with allergic indices and barrier-related markers support a close relationship between fecal metabolic profiles and host response-related parameters.

Integrated correlations among microbial taxa, metabolites, barrier markers, and allergic indices further support a coordinated host–microbe metabolic response associated with Gen pretreatment. Genera showing higher relative abundance in the H-Gen group were positively associated with metabolites such as indole-3-acrylic acid and guanosine, whereas taxa showing higher relative abundance in the OVA group were associated with metabolites linked to the allergic phenotype. These patterns suggest that Gen pretreatment was accompanied by coordinated changes in gut microbiota composition and fecal metabolic profiles. However, these findings remain mechanistically informative rather than definitive, as multi-omics correlations cannot establish direct causal relationships. Therefore, the metabolomic and integrative analyses should be interpreted as hypothesis-generating evidence for host–microbe metabolic pathways potentially involved in the response to Gen pretreatment.

Several limitations should be acknowledged. First, although associations were observed among immune regulation, intestinal barrier integrity, gut microbiota composition, SCFA profiles, and fecal metabolites, direct causal relationships were not established. Further studies using microbiota depletion, fecal microbiota transplantation, targeted metabolite supplementation, bacterial intervention, receptor-level validation, or cell-specific approaches are needed to verify the underlying pathways. Second, the evidence was obtained in a murine OVA-induced FA model, and caution is therefore required when extrapolating these findings to human FA, which is more heterogeneous in allergens, genetic background, disease severity, and clinical presentation. Third, although the BMDC experiments supported an immunomodulatory role for Gen, this in vitro model does not fully reproduce the intestinal mucosal environment, and the molecular targets underlying dendritic-cell-related effects remain to be clarified. Finally, purified commercial Gen rather than biomass-derived extracts was used in the present study. Therefore, the present findings cannot be directly extrapolated to complex Gen-containing plant extracts or biomass-derived preparations.

Accordingly, direct implications for biomass valorization remain speculative and require dedicated studies on source selection, extraction efficiency, compositional standardization, processing stability, bioavailability, and efficacy validation of real biomass-derived materials. Future work should compare purified Gen with standardized Gen-containing preparations and determine whether similar immune, barrier-related, microbiota-associated, and metabolic responses can be reproduced in application-oriented FA prevention models.

## 5. Conclusions

In conclusion, Gen pretreatment prevented OVA-induced allergic responses in mice and was associated with modulation of allergic immune responses, preservation of intestinal barrier-related markers, and coordinated changes in gut microbiota composition and fecal metabolic profiles. These findings provide preliminary mechanistic evidence suggesting that Gen may influence experimental FA through interconnected immune, epithelial barrier, and host–microbe metabolic pathways.

## Data Availability

The original contributions presented in the study are included in the article/Supplementary Materials; further inquiries can be directed to the corresponding author.

## Supplementary Materials

The following supporting information can be downloaded at https://www.mdpi.com/article/10.3390/foods15111995/s1, Figure S1: Representative flow cytometry plots of splenic CD4^+^ T-cell subsets in OVA-induced food allergy mice. (A–D) Intracellular staining for CD4^+^T-bet^+^ (Th1), CD4^+^GATA3^+^ (Th2), CD4^+^RORγt^+^ (Th17) and CD4^+^Foxp3^+^ (Treg) cells; Figure S2: Effects of genistein pretreatment on the relative mRNA expression levels of transcription factors and cytokines in jejunal tissue. (A–G) Relative mRNA expression levels of *T-bet*, *GATA3*, *RORγt*, *Foxp3*, *IL-4*, *IL-5* and *IFN-γ*; Figure S3: Effects of genistein on the viability of bone marrow-derived dendritic cells (BMDCs) assessed by CCK-8 assay; Figure S4: Representative flow cytometry plots of BMDC maturation after genistein treatment. (A–E) Surface expression of CD86, CD103, MHC-II, CD40 and CD80 on BMDCs; Figure S5: Representative flow cytometry plots of CD4^+^ T-cell polarization in co-cultures with genistein-treated BMDCs. (A–D) Intracellular expression of T-bet (Th1), GATA3 (Th2), RORγt (Th17), and Foxp3 (Treg) in CD4^+^ T cells; Figure S6: Effects of genistein pretreatment on gut microbiota α-diversity indices. (A) Sequencing coverage. (B) Observed species. (C) ACE index; Table S1: Sources and catalog numbers of reagents and equipment; Table S2: Scoring criteria for anaphylactic symptoms; Table S3: Fecal morphology scoring criteria; Table S4: Primer sequences used for qPCR.
