# Supplementary material for: Genistein Pretreatment Attenuates Ovalbumin-Induced Food Allergy in Mice with Intestinal Barrier Preservation and Modulation of Gut Microbiota and Metabolites
Source: Foods. 2026 Jun 3;15(11):1995. doi: 10.3390/foods15111995 (PMC13257282; doi:10.3390/foods15111995)
Supplement: Supplementary file 1 [file foods-15-01995-s001.zip › foods-4299983-supplementary/Supplementary Files/Supplementary Materials.docx]

**Supplementary Materials**

**Genistein Pretreatment Attenuates Ovalbumin-Induced Food Allergy in Mice with Intestinal Barrier Preservation and Modulation of Gut Microbiota and Metabolites**

Xiaomei Yi ^a,c,d^, Wen Deng ^a,c,d^, Kuan Gao ^a,c,d^, Xiaoying Ou ^a,c,d^, Keyu Tang ^a,c,d^, Qian Zeng ^a,c,d^, Yuanyuan Ni ^a,c,d^, Xiaohui Liang ^a,c,d^, Zhihua Wu ^a,b,c^, Yong Wu ^a,b^, Yanhai Xie ^a^, Hongbing Chen ^a,b,c^, Anshu Yang ^a,b,c*^

^a^ Sino-German Joint Research Institute, Nanchang University, Nanjing Dong Lu 235, Nanchang 330047, China

^b^ International Institute of Food Innovation, Nanchang University, Nanchang 330200, China

^c^ State Key Laboratory of Food Science and Resources, Nanchang University, Nanjing Dong Lu 235, Nanchang 330047, China

^d^ School of Food Science and Technology, Nanchang University, Nanchang 330047, China

^*^Corresponding author. Tel.: +86 791 88333529; Fax: +86 791 88333708.

E-mail: yanganshu@ncu.edu.cn (Anshu Yang)


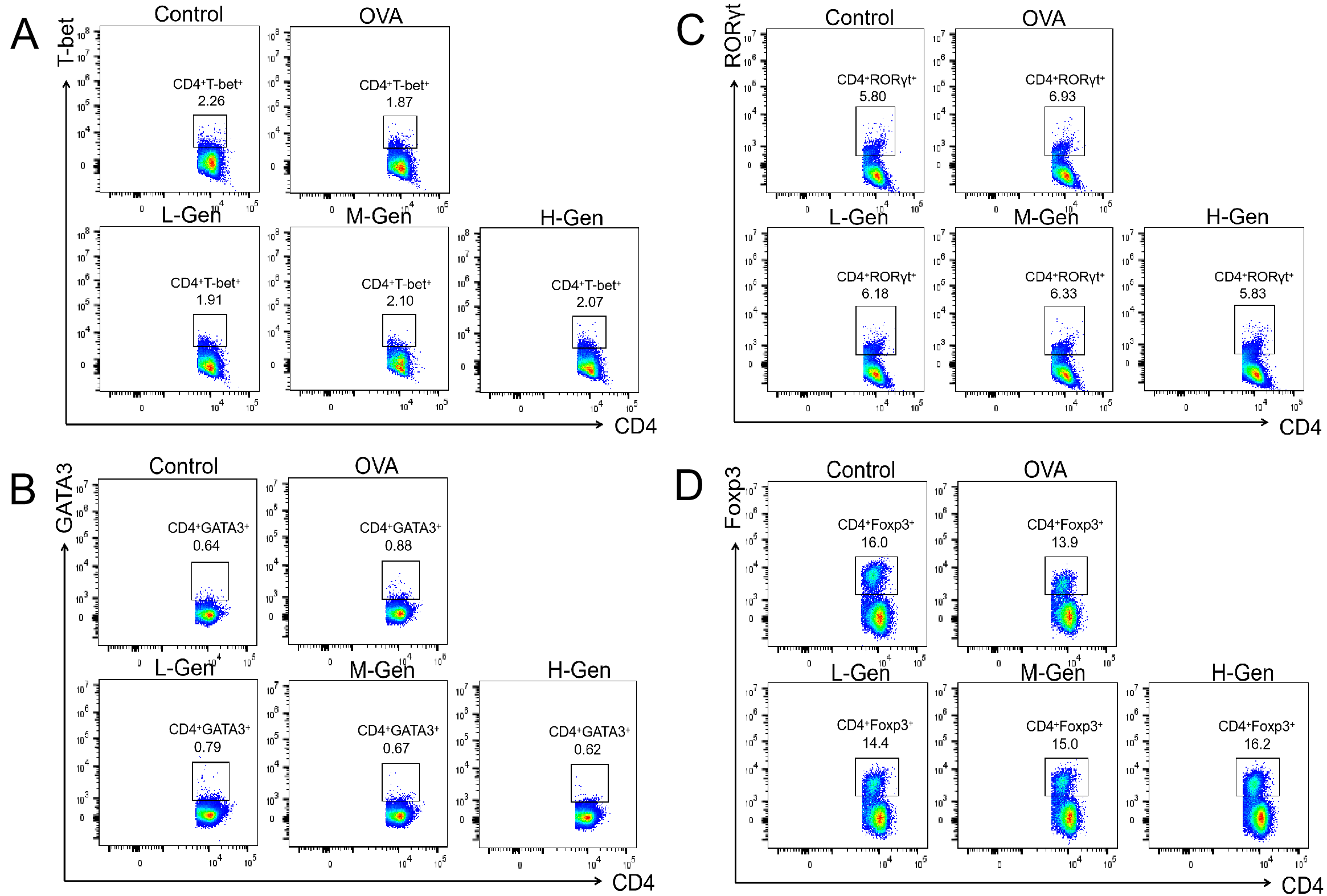


**Figure S1.** Representative flow cytometry plots of splenic CD4⁺ T-cell subsets in OVA-induced food allergy mice. (A–D) Intracellular staining for CD4⁺T-bet⁺ (Th1), CD4⁺GATA3⁺ (Th2), CD4⁺RORγt⁺ (Th17) and CD4⁺Foxp3⁺ (Treg) cells.


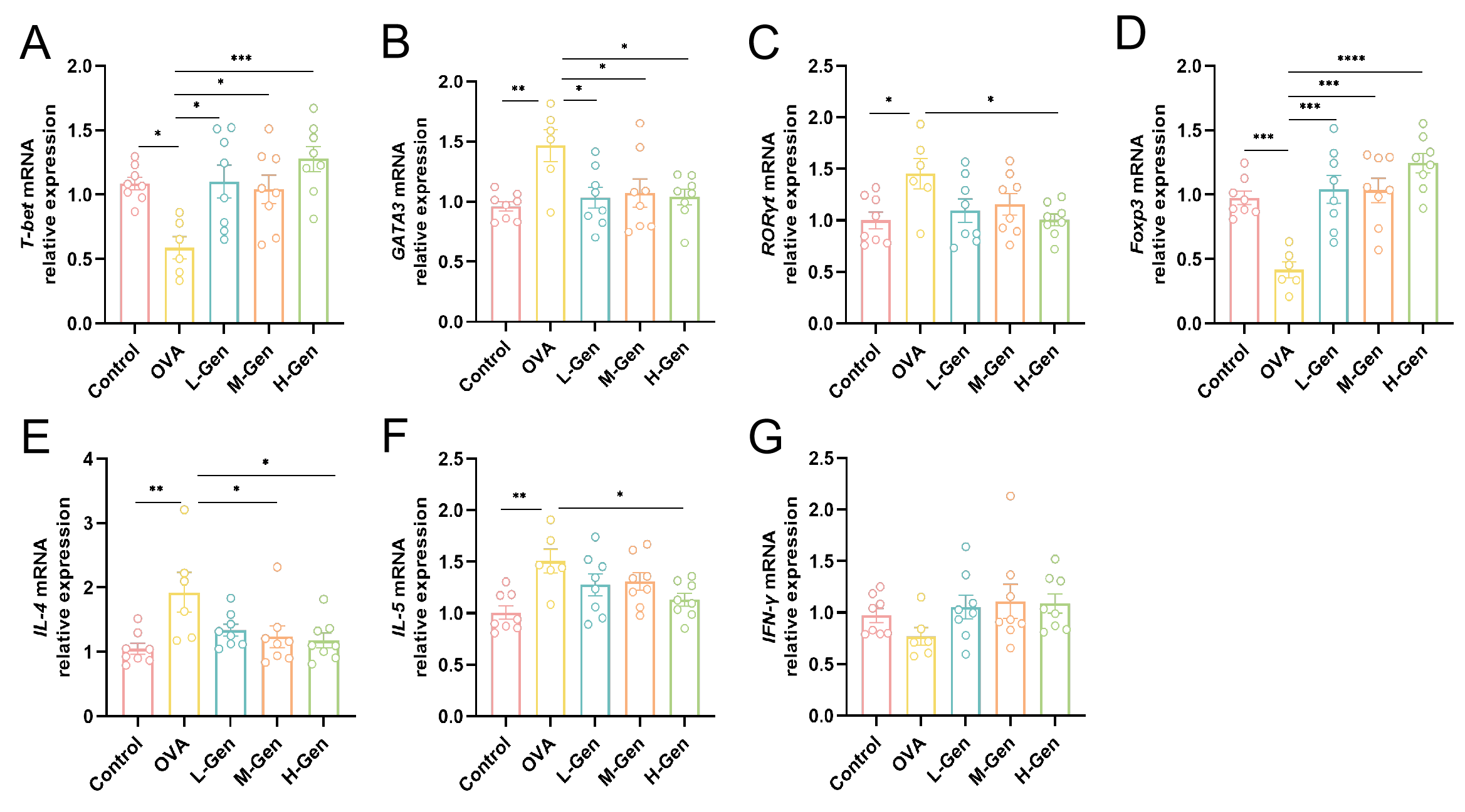


**Figure S2.** Effects of genistein pretreatment on the relative mRNA expression levels of transcription factors and cytokines in jejunal tissue. (A–G) Relative mRNA expression levels of *T-bet*, *GATA3*, *RORγt*, *Foxp3*, *IL-4*, *IL-5* and *IFN-γ*.


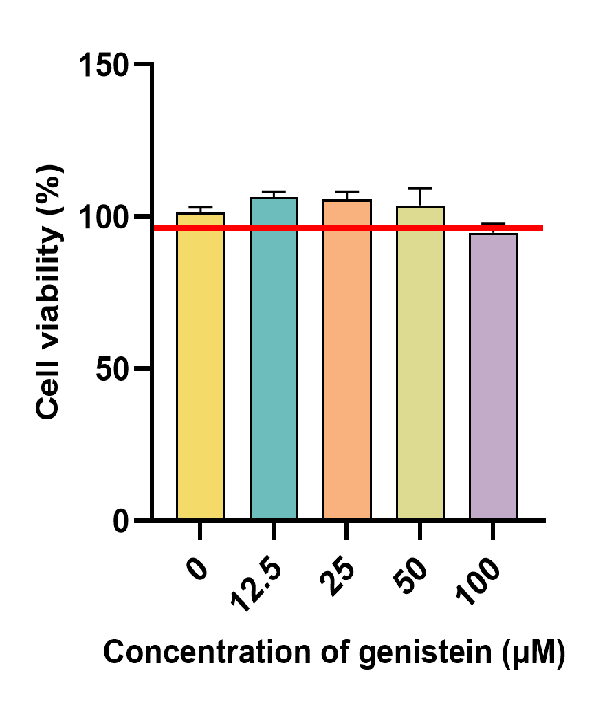


**Figure S3.** Effects of genistein on the viability of bone marrow–derived dendritic cells (BMDCs) assessed by CCK-8 assay.


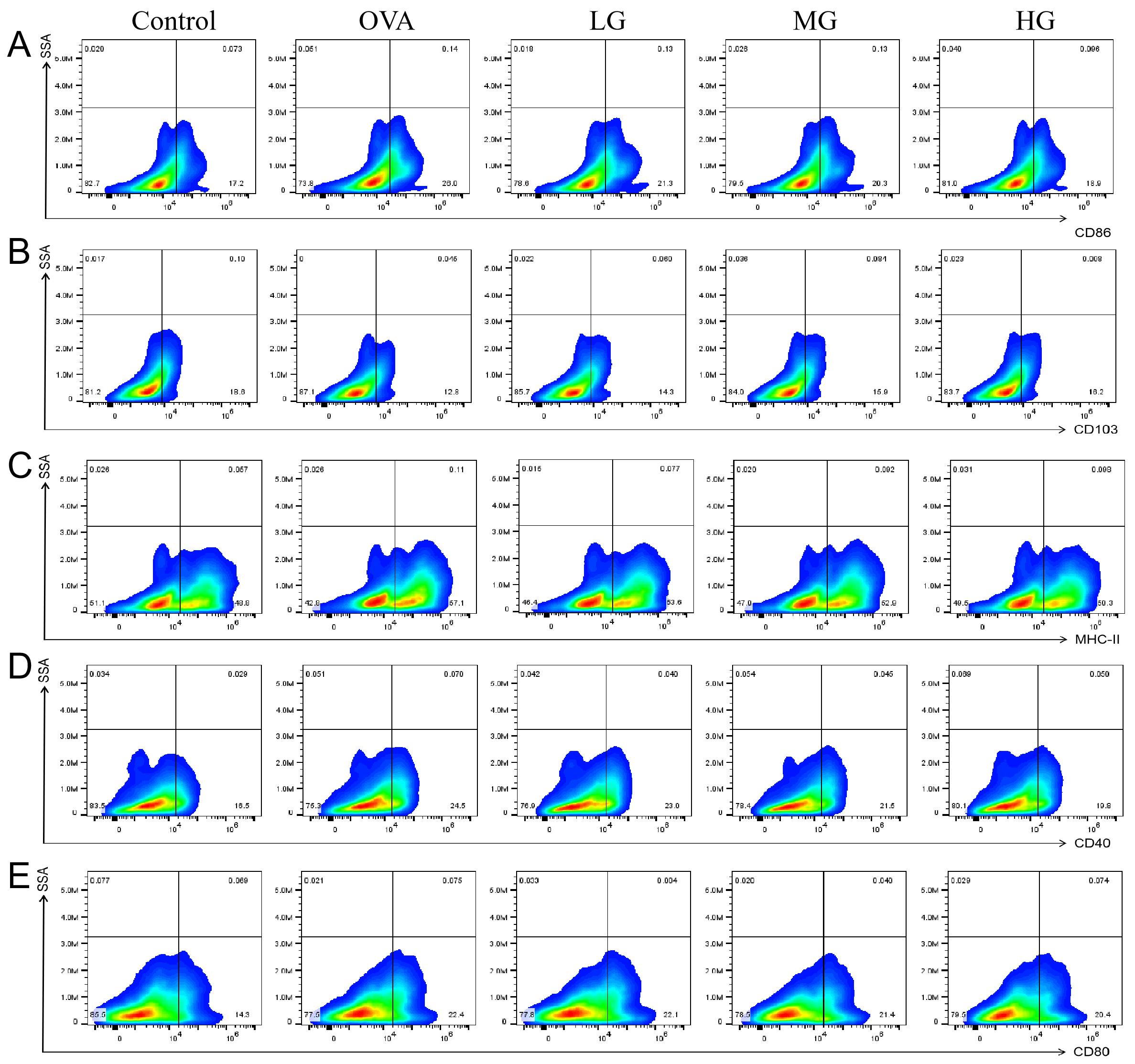


**Figure S4.** Representative flow cytometry plots of BMDC maturation after genistein treatment. (A–E) Surface expression of CD86, CD103, MHC-II, CD40 and CD80 on BMDCs.

**
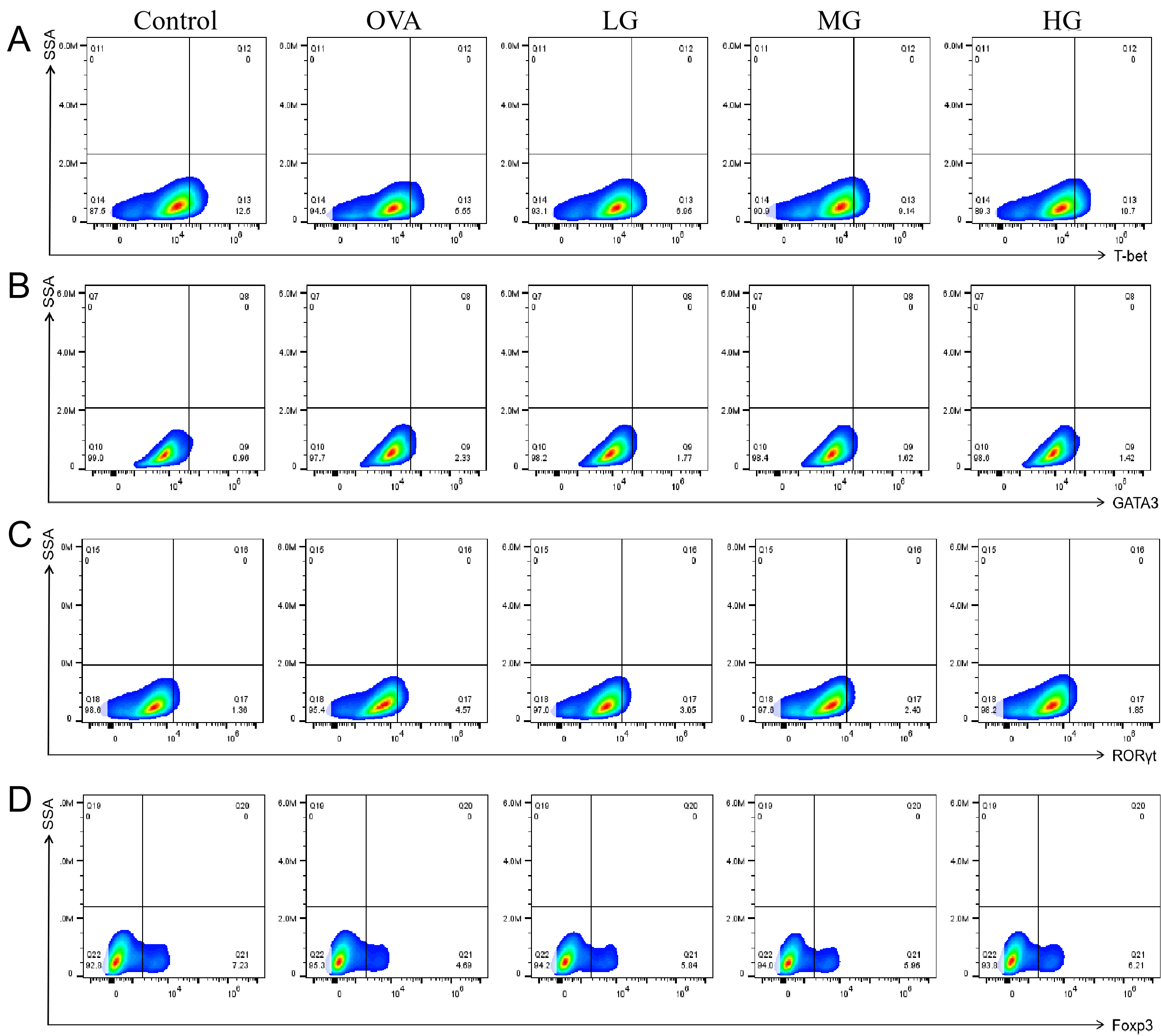
**

**Figure S5.** Representative flow cytometry plots of CD4^+^ T-cell polarization in co-cultures with genistein-treated BMDCs. (A–D) Intracellular expression of T-bet (Th1), GATA3 (Th2), RORγt (Th17), and Foxp3 (Treg) in CD4⁺ T cells.


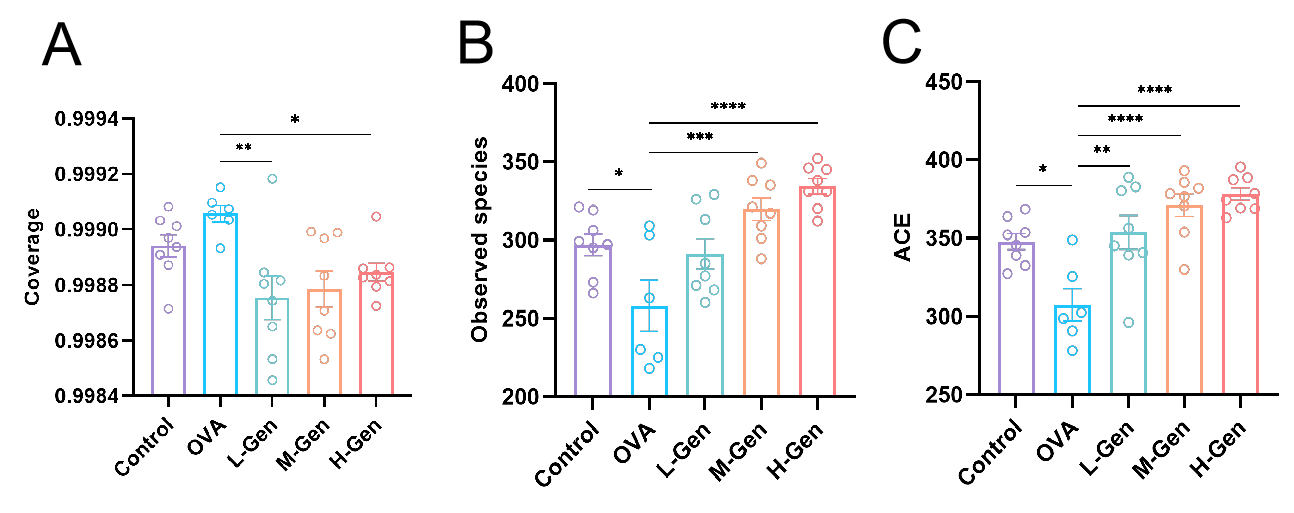


**Figure S6.** Effects of genistein pretreatment on gut microbiota α-diversity indices. (A) Sequencing coverage. (B) Observed species. (C) ACE index.

**Table S1.** Sources and catalog numbers of reagents and equipment.

| **Reagent Name** | **Vendor** | **Catalog No.** |
| --- | --- | --- |
| OVA peptide | MedChemExpres | HY-P0286 |
| IgE | SouthernBiotech | 1110-08 |
| IgG | SouthernBiotech | 6120-01 |
| IgG1 | SouthernBiotech | 1071-08 |
| IgG2a | SouthernBiotech | 1081-05 |
| mMCP-1 | Thermo Fisher Scientific | 88-7503-22 |
| IL-4 | Thermo Fisher Scientific | 88-7044-22 |
| IL-5 | Thermo Fisher Scientific | 88-7054-22 |
| IL-13 | Thermo Fisher Scientific | 88-7137-22 |
| IL-17A | Thermo Fisher Scientific | 88-7371-22 |
| IFN-γ | Thermo Fisher Scientific | 88-7314-22 |
| IL-10 | Thermo Fisher Scientific | 88-7105-22 |
| Ovalbumin-FITC | Ruixi Biological Technology Co., Ltd. | R-OF-001 |
| Anti-CD4 | BioLegend | 100431 |
| Anti-CD25 | BioLegend | 102015 |
| Anti-CD40 | BioLegend | 124622 |
| Anti-CD86 | BioLegend | 105008 |
| Anti-CD80 | BioLegend | 104707 |
| Anti-MHC-II | BioLegend | 107631 |
| Anti-CD11c | BioLegend | 117309 |
| Anti-CD103 | BioLegend | 121433 |
| Fixation Buffer | BioLegend | 420801 |
| Perm/Wash Buffer | BioLegend | 421002 |
| Anti-T-bet | BioLegend | 644832 |
| Anti-GATA3 | BioLegend | 653803 |
| Anti-Foxp3 | BioLegend | 320013 |
| Anti-RORγt | BD Biosciences | 564722 |

**T****able S2.** Scoring criteria for anaphylactic symptoms.

| **Score** | **Symptoms** |
| --- | --- |
| 0 | No symptoms |
| 1 | Scratching and rubbing around the nose and head |
| 2 | Swelling around the eyes and mouth |
| 3 | Wheezing, labored respiration, or cyanosis around the mouth and tail |
| 4 | No activity after stimulation, tremor, or convulsions |
| 5 | Death |

**Table S3.** Fecal morphology scoring criteria.

| **Fecal morphology** | **Score** |
| --- | --- |
| Solid stool | 0 |
| Funicular stool | 1 |
| Slurry stool | 2 |
| Watery stool | 3 |

**Table S4.** Primer sequences used for qPCR.

| **Gene** | **Forward primer (5' to 3')** | **Reverse primer (5' to 3')** |
| --- | --- | --- |
| *Claudin-1* | GCCTTGATGGTAATTGGCATCC | GGCCACTAATGTCGCCAGAC |
| *Occludin* | TTGAAAGTCCACCTCCTTACAGA | CCGGATAAAAAGAGTACGCTGG |
| *ZO-1* | CGAGGCATCATCCCAAATAAGAAC | TCCAGAAGTCTGCCCGATCAC |
| *IL-4* | CTCATGGAGCTGCAGAGACTCTT | CATTCATGGTGCAGCTTATCGA |
| *IL-5* | AACCCTGAGTTTCAGGACTCGCCTT | TCTTCAGCGCTGGCCTTCAGCAA |
| *IFN-γ* | ATGAACGCTACACACTGCATC | CCATCCTTTTGCCAGTTCCTC |
| *T-bet* | CTGCCTACCAGAACGCAGA | AAACGGCTGGGAACAGGA |
| *GATA3* | TTATCAAGCCCAAGCGAAG | CCATTAGCGTTCCTCCTCCA |
| *RORγt* | ACAAATTGAAGTGATCCCTTGC | GGAGTAGGCCACATTACACTG |
| *Foxp3* | CCACGGGCACTATCACACAT | TTGCTTGAGGCTGCGTATGA |
| *GAPDH* | CCTGTTGCTGTAGCCGTATTCA | CCAGGTTGTCTCCTGCGACTT |
