# Supplementary material for: Genistein Pretreatment Attenuates Ovalbumin-Induced Food Allergy in Mice with Intestinal Barrier Preservation and Modulation of Gut Microbiota and Metabolites
Source: Foods. 2026 Jun 3;15(11):1995. doi: 10.3390/foods15111995 (PMC13257282; doi:10.3390/foods15111995)
Supplement: Supplementary file 1 [file foods-15-01995-s001.zip › foods-4299983-supplementary/Supplementary Files/Table S1.docx]

**Table S1.** Sources and catalog numbers of reagents and equipment.

| **Reagent Name** | **Vendor** | **Catalog No.** |
| --- | --- | --- |
| OVA peptide | MedChemExpres | HY-P0286 |
| IgE | SouthernBiotech | 1110-08 |
| IgG | SouthernBiotech | 6120-01 |
| IgG1 | SouthernBiotech | 1071-08 |
| IgG2a | SouthernBiotech | 1081-05 |
| mMCP-1 | Thermo Fisher Scientific | 88-7503-22 |
| IL-4 | Thermo Fisher Scientific | 88-7044-22 |
| IL-5 | Thermo Fisher Scientific | 88-7054-22 |
| IL-13 | Thermo Fisher Scientific | 88-7137-22 |
| IL-17A | Thermo Fisher Scientific | 88-7371-22 |
| IFN-γ | Thermo Fisher Scientific | 88-7314-22 |
| IL-10 | Thermo Fisher Scientific | 88-7105-22 |
| Ovalbumin-FITC | Ruixi Biological Technology Co., Ltd. | R-OF-001 |
| Anti-CD4 | BioLegend | 100431 |
| Anti-CD25 | BioLegend | 102015 |
| Anti-CD40 | BioLegend | 124622 |
| Anti-CD86 | BioLegend | 105008 |
| Anti-CD80 | BioLegend | 104707 |
| Anti-MHC-II | BioLegend | 107631 |
| Anti-CD11c | BioLegend | 117309 |
| Anti-CD103 | BioLegend | 121433 |
| Fixation Buffer | BioLegend | 420801 |
| Perm/Wash Buffer | BioLegend | 421002 |
| Anti-T-bet | BioLegend | 644832 |
| Anti-GATA3 | BioLegend | 653803 |
| Anti-Foxp3 | BioLegend | 320013 |
| Anti-RORγt | BD Biosciences | 564722 |
