# Supplementary material for: Genistein Pretreatment Attenuates Ovalbumin-Induced Food Allergy in Mice with Intestinal Barrier Preservation and Modulation of Gut Microbiota and Metabolites
Source: Foods. 2026 Jun 3;15(11):1995. doi: 10.3390/foods15111995 (PMC13257282; doi:10.3390/foods15111995)
Supplement: Supplementary file 1 [file foods-15-01995-s001.zip › foods-4299983-supplementary/Supplementary Files/Table S2.docx]

**T****able S2.** Scoring criteria for anaphylactic symptoms

| **Score** | **Symptoms** |
| --- | --- |
| 0 | No symptoms |
| 1 | Scratching and rubbing around the nose and head |
| 2 | Swelling around the eyes and mouth |
| 3 | Wheezing, labored respiration, or cyanosis around the mouth and tail |
| 4 | No activity after stimulation, tremor, or convulsions |
| 5 | Death |
