# Supplementary material for: Genistein Pretreatment Attenuates Ovalbumin-Induced Food Allergy in Mice with Intestinal Barrier Preservation and Modulation of Gut Microbiota and Metabolites
Source: Foods. 2026 Jun 3;15(11):1995. doi: 10.3390/foods15111995 (PMC13257282; doi:10.3390/foods15111995)
Supplement: Supplementary file 1 [file foods-15-01995-s001.zip › foods-4299983-supplementary/Supplementary Files/Table S3.docx]

**Table S3.** Fecal morphology scoring criteria

| **Fecal morphology** | **Score** |
| --- | --- |
| Solid stool | 0 |
| Funicular stool | 1 |
| Slurry stool | 2 |
| Watery stool | 3 |
