# Supplementary material for: Genistein Pretreatment Attenuates Ovalbumin-Induced Food Allergy in Mice with Intestinal Barrier Preservation and Modulation of Gut Microbiota and Metabolites
Source: Foods. 2026 Jun 3;15(11):1995. doi: 10.3390/foods15111995 (PMC13257282; doi:10.3390/foods15111995)
Supplement: Supplementary file 1 [file foods-15-01995-s001.zip › foods-4299983-supplementary/Supplementary Files/Table S4.docx]

**Table S4.** Primer sequences used for qPCR

| **Gene** | **Forward primer (5' to 3')** | **Reverse primer (5' to 3')** |
| --- | --- | --- |
| *Claudin-1* | GCCTTGATGGTAATTGGCATCC | GGCCACTAATGTCGCCAGAC |
| *Occludin* | TTGAAAGTCCACCTCCTTACAGA | CCGGATAAAAAGAGTACGCTGG |
| *ZO-1* | CGAGGCATCATCCCAAATAAGAAC | TCCAGAAGTCTGCCCGATCAC |
| *IL-4* | CTCATGGAGCTGCAGAGACTCTT | CATTCATGGTGCAGCTTATCGA |
| *IL-5* | AACCCTGAGTTTCAGGACTCGCCTT | TCTTCAGCGCTGGCCTTCAGCAA |
| *IFN-γ* | ATGAACGCTACACACTGCATC | CCATCCTTTTGCCAGTTCCTC |
| *T-bet* | CTGCCTACCAGAACGCAGA | AAACGGCTGGGAACAGGA |
| *GATA3* | TTATCAAGCCCAAGCGAAG | CCATTAGCGTTCCTCCTCCA |
| *RORγt* | ACAAATTGAAGTGATCCCTTGC | GGAGTAGGCCACATTACACTG |
| *Foxp3* | CCACGGGCACTATCACACAT | TTGCTTGAGGCTGCGTATGA |
| *GAPDH* | CCTGTTGCTGTAGCCGTATTCA | CCAGGTTGTCTCCTGCGACTT |
