# Supplementary figures and images for: Genistein Pretreatment Attenuates Ovalbumin-Induced Food Allergy in Mice with Intestinal Barrier Preservation and Modulation of Gut Microbiota and Metabolites
Source: Foods. 2026 Jun 3;15(11):1995. doi: 10.3390/foods15111995 (PMC13257282; doi:10.3390/foods15111995)

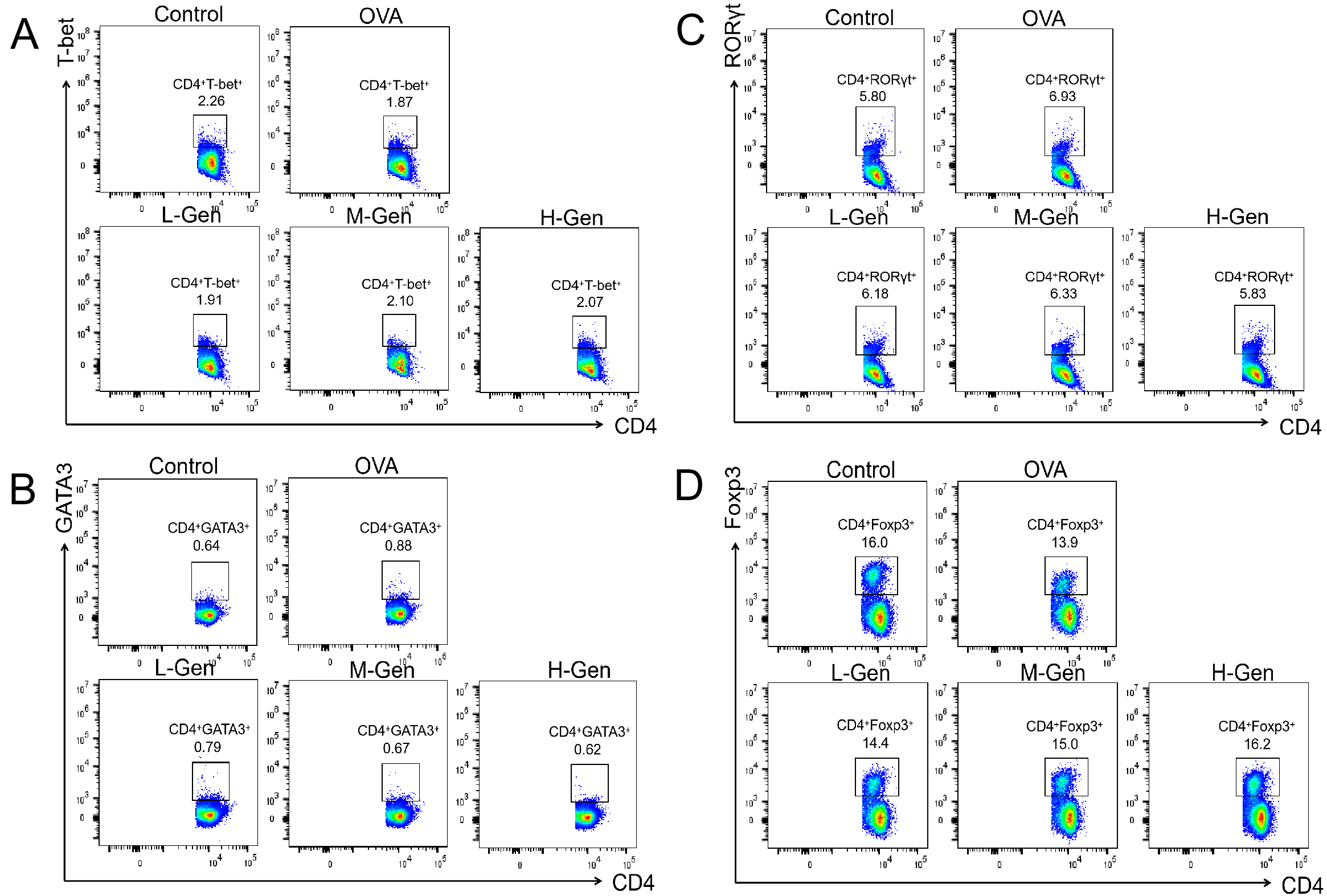

Supplement: Supplementary file 1 [file foods-15-01995-s001.zip › foods-4299983-supplementary/Supplementary Files/Figure S1.tif]

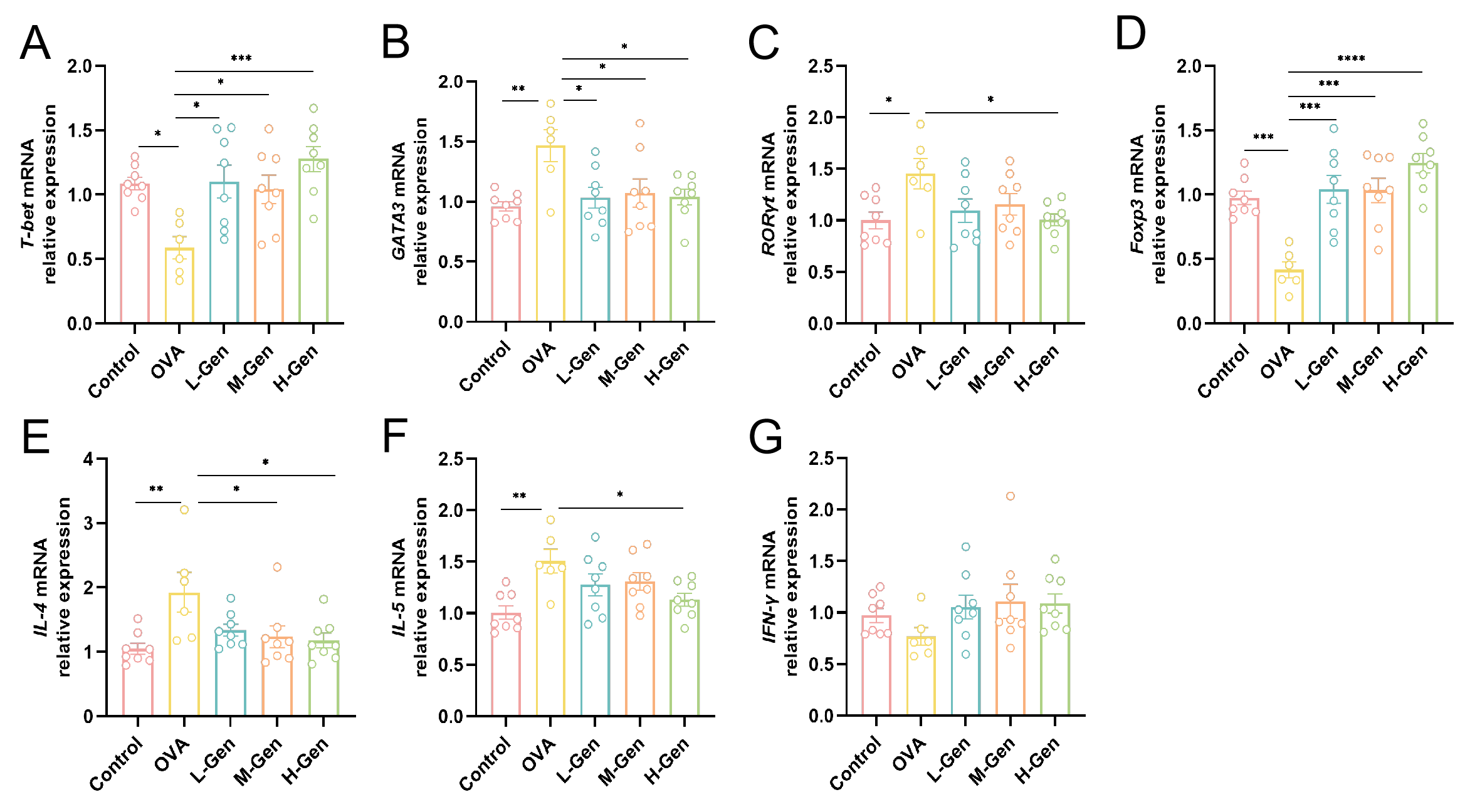

Supplement: Supplementary file 1 [file foods-15-01995-s001.zip › foods-4299983-supplementary/Supplementary Files/Figure S2.tif]

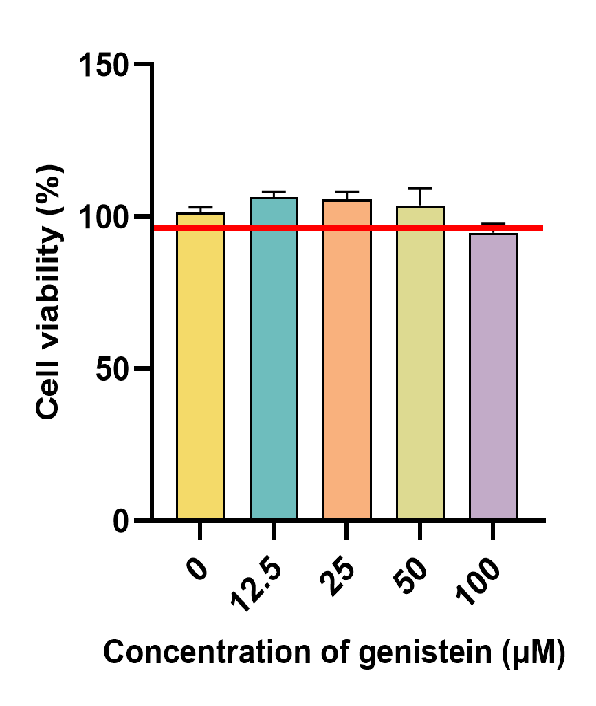

Supplement: Supplementary file 1 [file foods-15-01995-s001.zip › foods-4299983-supplementary/Supplementary Files/Figure S3.tif]

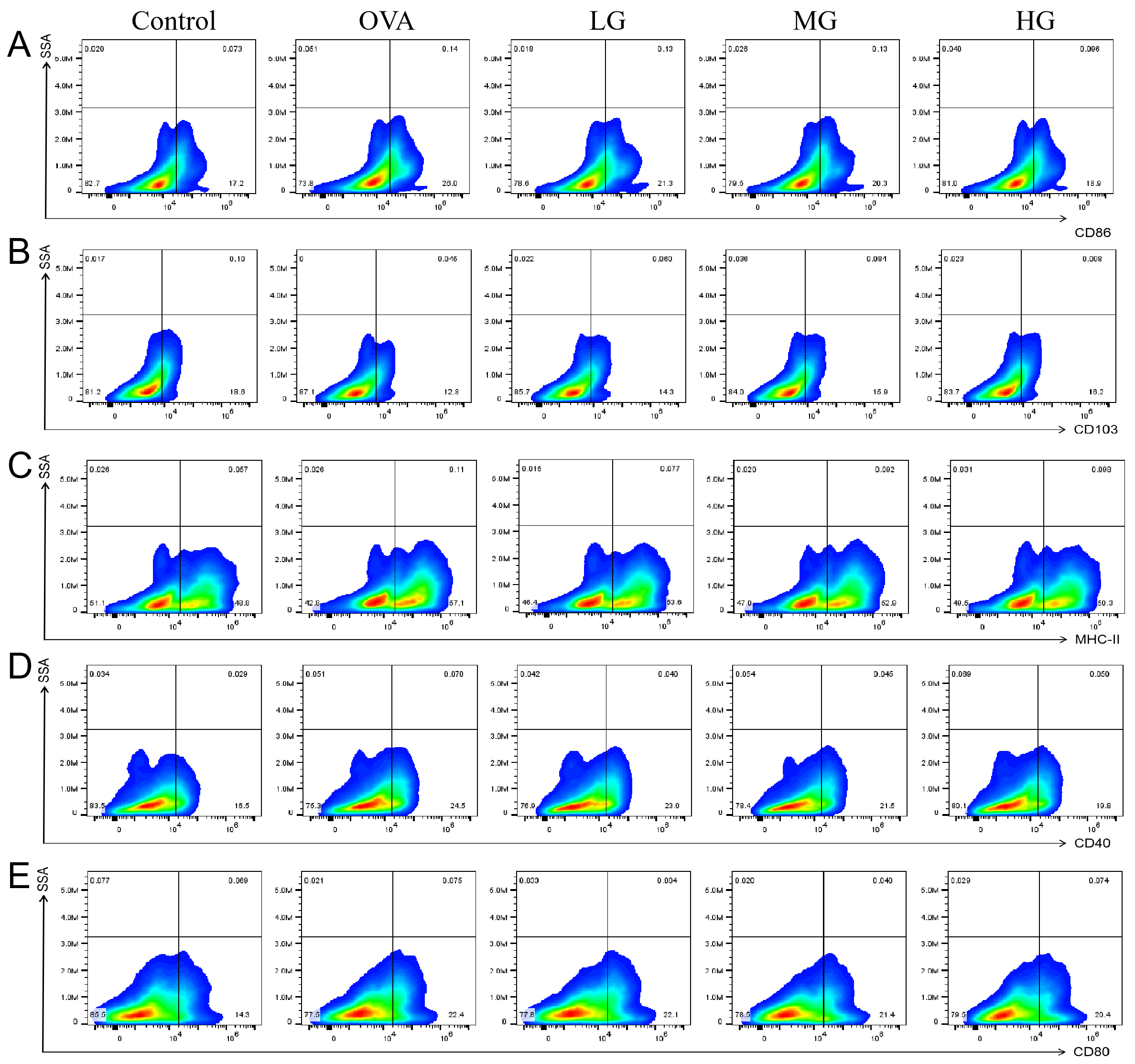

Supplement: Supplementary file 1 [file foods-15-01995-s001.zip › foods-4299983-supplementary/Supplementary Files/Figure S4.tif]

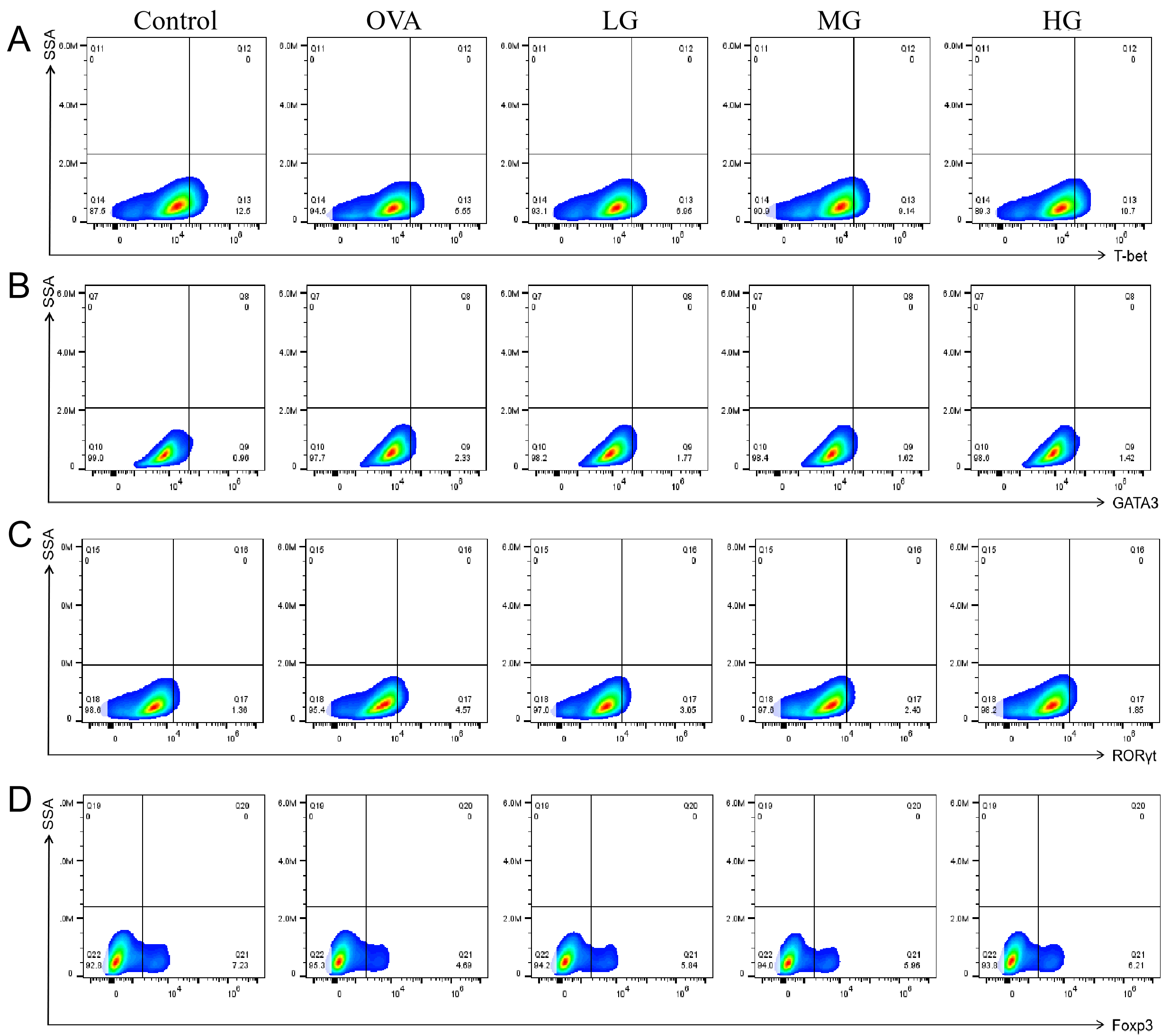

Supplement: Supplementary file 1 [file foods-15-01995-s001.zip › foods-4299983-supplementary/Supplementary Files/Figure S5.tif]

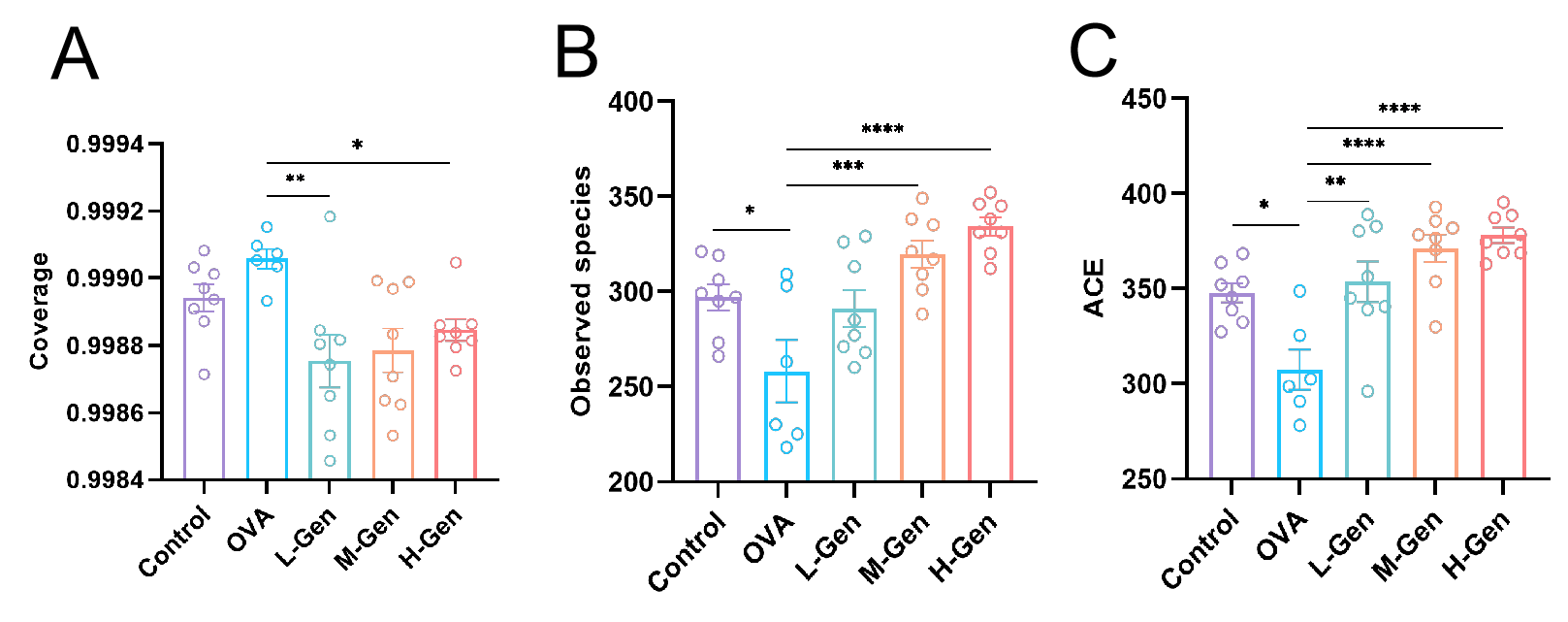

Supplement: Supplementary file 1 [file foods-15-01995-s001.zip › foods-4299983-supplementary/Supplementary Files/Figure S6.tif]
